# Concurrent Strength and Endurance Training: A Systematic Review and Meta-Analysis on the Impact of Sex and Training Status

**DOI:** 10.1007/s40279-023-01943-9

**Published:** 2023-10-17

**Authors:** Raven O. Huiberts, Rob C. I. Wüst, Stephan van der Zwaard

**Affiliations:** 1 Department of Human Movement Sciences, Faculty of Behavioural and Movement Sciences, Vrije Universiteit Amsterdam, Amsterdam Movement Sciences, Amsterdam, The Netherlands; 2 Department of Cardiology, Amsterdam University Medical Center, University of Amsterdam, Meibergdreef 9, 1105 AZ Amsterdam, The Netherlands

## Abstract

**Background:**

Many sports require maximal strength and endurance performance. Concurrent strength and endurance training can lead to suboptimal training adaptations. However, how adaptations differ between males and females is currently unknown. Additionally, current training status may affect training adaptations.

**Objective:**

We aimed to assess sex-specific differences in adaptations in strength, power, muscle hypertrophy, and maximal oxygen consumption ($\dot{V}$O_2max_) to concurrent strength and endurance training in healthy adults. Second, we investigated how training adaptations are influenced by strength and endurance training status.

**Methods:**

A systematic review and meta-analysis was conducted according to PRISMA (Preferred Reporting Items for Systematic Reviews and Meta-Analyses) guidelines, and a Cochrane risk of bias was evaluated. ISI Web of science, PubMed/MEDLINE, and SPORTDiscus databases were searched using the following inclusion criteria: healthy adults aged 18–50 years, intervention period of ≥ 4 weeks, and outcome measures were defined as upper- and lower-body strength, power, hypertrophy, and/or $\dot{V}$O_2max_. A meta-analysis was performed using a random-effects model and reported in standardized mean differences.

**Results:**

In total, 59 studies with 1346 participants were included. Concurrent training showed blunted lower-body strength adaptations in males, but not in females (male: − 0.43, 95% confidence interval [− 0.64 to − 0.22], female: 0.08 [− 0.34 to 0.49], group difference: *P* = 0.03). No sex differences were observed for changes in upper-body strength (*P* = 0.67), power (*P* = 0.37), or $\dot{V}$O_2max_ (*P* = 0.13). Data on muscle hypertrophy were insufficient to draw any conclusions. For training status, untrained but not trained or highly trained endurance athletes displayed lower $\dot{V}$O_2max_ gains with concurrent training (*P* = 0.04). For other outcomes, no differences were found between untrained and trained individuals, both for strength and endurance training status.

**Conclusions:**

Concurrent training results in small interference for lower-body strength adaptations in males, but not in females. Untrained, but not trained or highly trained endurance athletes demonstrated impaired improvements in $\dot{V}$O_2max_ following concurrent training. More studies on females and highly strength-trained and endurance-trained athletes are warranted.

**Clinical Trial Registration:**

PROSPERO: CRD42022370894.

**Supplementary Information:**

The online version contains supplementary material available at 10.1007/s40279-023-01943-9.

## Key Points

**Table:** 

Concurrent training resulted in blunted lower-body strength adaptations in males, but not in females.
For training status, untrained but not trained or highly trained endurance athletes displayed impaired improvements for maximal oxygen consumption with concurrent training.
Most concurrent training studies include relatively untrained participants, and highly trained athletes are under-represented in the literature.
Training status should be considered both in terms of strength and endurance training status and can be evaluated according to the mentioned classification framework.

## Introduction

When it comes to optimal physical performance, there are few sports that exclusively require a high maximal strength or a high endurance performance. Instead, many sports demand a combination of a high maximal strength and endurance performance [1, 2]. For this reason, athletes are encouraged to perform concurrent training, which combines both strength and endurance within their training program. Simultaneously training for strength and endurance is not without limitations when it comes to optimizing training adaptations. First, skeletal muscles of strength- and endurance-trained individuals are specifically trained, focused either on increasing muscle size and neuromuscular adaptations or on oxygen delivery and utilization. However, the extremes of these two traits are physiologically incompatible, as there is an inverse relationship between muscle fiber cross-sectional area and mitochondrial oxidative capacity [3–5]. Second, concurrent training potentially leads to blunted strength, power, or hypertrophic adaptations compared with strength training alone. This ‘interference effect’ was first reported in Hickson’s seminal publication on concurrent training in 1980 [6]. Since then, the literature on concurrent training has expanded. Recently, an updated meta-analysis [7] concluded that power gains were impaired with concurrent strength and endurance training, but that hypertrophy and maximal strength development were not compromised. Others [8] showed that maximal oxygen consumption ($\dot{V}$O_2max_) gains were also not compromised by concurrent training.

The magnitude of the interference effect with concurrent training may be influenced by inter-individual variations, differences in experimental design, training intervention, and the type of outcome measure. Many of these potentially contributing factors have been analyzed before [7–10]. One important inter-individual factor that has received very little attention in the literature is the differences in training responses between males and females. Sex-related adaptations with concurrent training may occur, owing to differences in endocrine and muscle physiology, or physical performance. On average, males have larger muscle fibers, more skeletal muscle mass, and greater strength [11–13], while females tend to have slower contractile properties [14]. Males tend to have greater improvements in absolute muscle size, strength, and power compared with females [15, 16], but females showed larger relative increases in upper-body strength than males [17]. Both sexes show similar relative increases in lower-body strength and hypertrophy after strength training. *Grosso modo*, males tend to have a higher $\dot{V}$O_2max_, even after normalization to body mass or fat-free mass [18–20], likely because females are generally smaller and have a lower cardiac output and stroke volume, hemoglobin levels, and blood volume [18–21]. Following endurance training, the absolute and relative increases in $\dot{V}$O_2max_ were also larger in males compared with females [22]. It is therefore conceivable that there may be sex differences in the adaptations to concurrent training.

Another inter-individual variation includes the current training status of the participant, which may significantly impact the magnitude of the interference effect with concurrent training. Coffey and Hawley [23] hypothesized that adaptations are more compromised following concurrent training in subjects with a longer training history, i.e., will be more pronounced in highly trained compared with untrained participants. Accordingly, elite athletes of sports that require both high sprint/peak power and endurance (e.g., rowing and cycling) indeed seem to experience that optimizing both traits at the same time is more difficult, as highlighted by the inverse relationships between athletes’ sprint/peak power and endurance performance/$\dot{V}$O_2max_ [3, 24]. However, previous meta-analyses on concurrent training did not find any differences in adaptations between different levels of training status for maximal strength, power, and hypertrophy [7, 9, 10], when there was sufficient time to recover between strength and endurance training sessions. In addition, for adaptations of $\dot{V}$O_2max_ with concurrent training, the effect of training status has not yet been assessed. It should be noted that training status is generally considered to be one-dimensional: a participant is untrained, trained, or highly trained. However, this neglects the fact that athletes can optimize for both their strength and endurance capacities. For example, professional marathon runners are highly trained endurance athletes, but relatively untrained for maximal absolute strength. Importantly, prior meta-analyses on concurrent training did not distinguish between both endurance- and strength-trained status [7, 9, 10, 25].

The aim of this study was to assess sex-specific differences in adaptations to concurrent strength and endurance training for strength, power, muscle hypertrophy, and $\dot{V}$O_2max_ in healthy adults. Second, we investigated how adaptations to concurrent training depend on strength and endurance training status. Such a systematic review and meta-analysis not only provides more insight into the concurrent training effects of various populations, but also highlights which populations may be under-represented in the concurrent training literature.

## Methods

### Systematic Literature Search

**Fig. 1:**
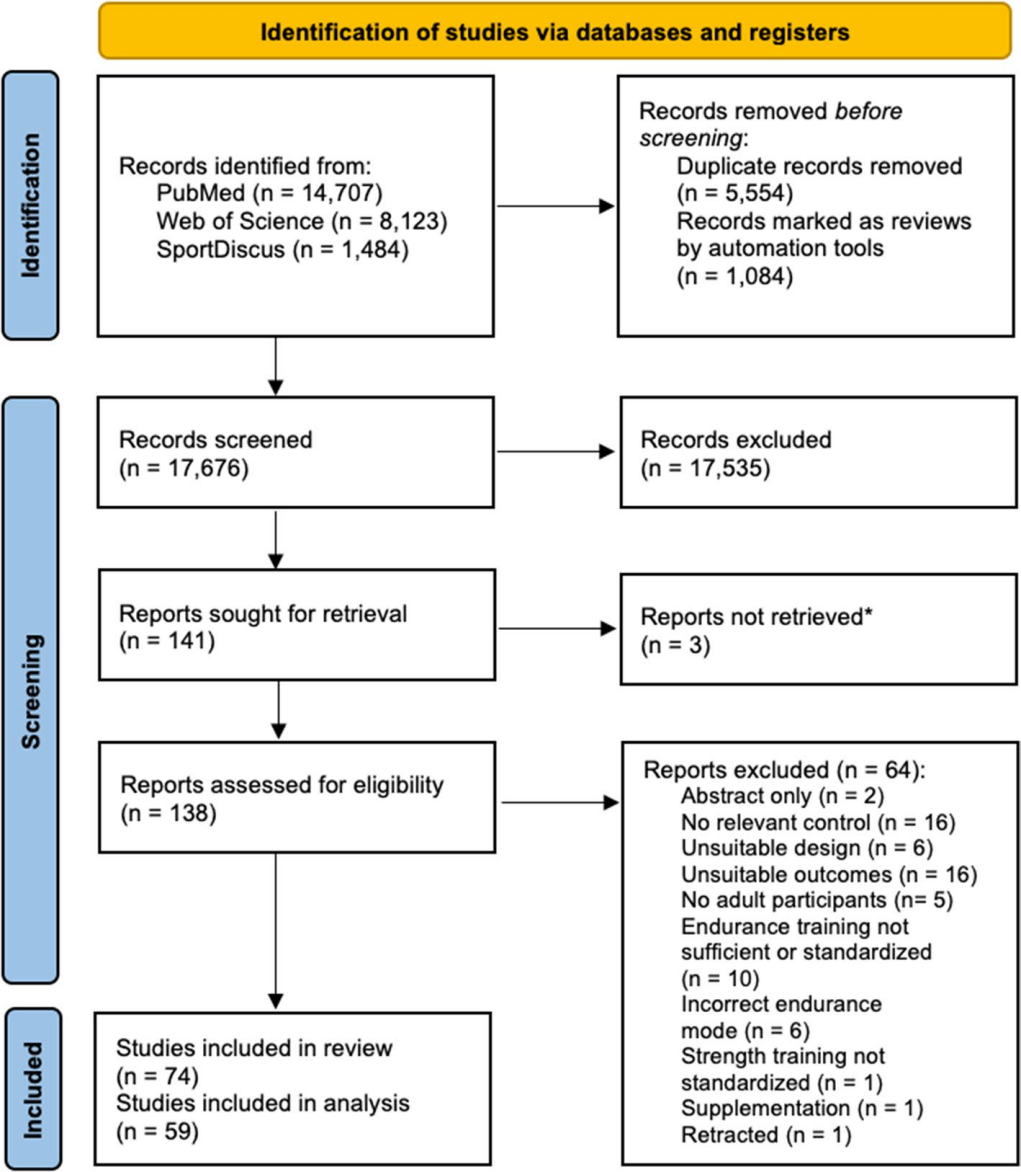
Flowchart of data search and selection of studies. *From three studies, no full text could be obtained

A systematic literature search was conducted according to the PRISMA (Preferred Reporting Items for Systematic Reviews and Meta-Analyses) guidelines, and registered with the International Database of Prospectively Registered Systematic Reviews in Health and Social Care (PROSPERO, CRD42022370894). The ISI Web of science, PubMed/MEDLINE, and SPORTDiscus databases were searched on 14 November, 2022, using the following search string: ((“Concurrent training’’ OR “Combined training’’) OR ((“Endurance training’’ OR “Aerobic training’’) AND (“Resistance training” OR “Strength training’’ OR “Weight training’’)) AND (“Maximal strength’’ OR “1RM’’ OR “One repetition max’’ OR “Explosive strength’’ OR “Counter movement jump’’ OR “Squat jump’’ OR “Wingate test’’ OR “Peak power’’ OR “Hypertrophy’’ OR “Cross-sectional area’’ OR “Muscle thickness’’ OR “VO2max’’ OR “VO2peak’’ OR “Aerobic capacity’’ OR “Maximal oxygen uptake’’ OR “Maximal oxygen consumption’’)). Only studies from 1980 or later were included, which was after the seminal paper on concurrent training by Hickson [6]. Almost all eligible studies were identified by PubMed/MEDLINE (95%), and only a very small portion by WoS (4%) or SPORTDiscus (1%). Retrieved titles from the search were saved and both duplicates and review articles were removed using automated tools (i.e., custom-written R scripts, R version 4.2.2). Titles and abstracts of all remaining studies were screened individually by two reviewers (RH and SZ). If the reviewers could not reach consensus, a third reviewer was consulted (RW). The search process and selection of studies are summarized in the flowchart in Fig. 1.

### Eligibility Criteria

Studies were included based on the PICO (Population, Intervention, Control and Outcomes) criteria [26]. The population included healthy male and female adults aged between 18 and 50 years. The intervention consisted of concurrent strength and endurance training for ≥ 4 weeks, where endurance training included cycling, cross-country skiing, swimming, rowing, or running and strength training was designed to improve strength, power, and/or hypertrophy, including concentric, eccentric, and plyometric training. Concurrent training groups were compared to an endurance training-only group (for $\dot{V}$O_2max_) or a strength training-only group (for other outcomes). Outcome measures related to maximal lower-body and upper-body strength, power, muscle hypertrophy, and $\dot{V}$O_2max_ (Table 1). For maximal strength, one-repetition maximum (1-RM) and isometric measurements of lower and upper extremities were considered. For power, jump tests and dynamic power tests (e.g., Wingate test) were evaluated. For muscle hypertrophy, muscle thickness or whole-muscle cross-sectional area was measured objectively using ultrasound, magnetic resonance imaging, or computed tomography. Studies were excluded if participants experienced injuries or an illness or if they used ergogenic aids or other sport-enhancing supplements (except for proteins and vitamins).

**Table 1:** Effects of concurrent training are evaluated for the following outcome measures. If multiple measurements exist for the same outcome measure, measurements are analyzed according to the presented hierarchy

Outcome measures	Measurements
Maximal lower-body strength	1-RM leg press (kg) 1-RM squat (kg) 1-RM half squat (kg)
Maximal upper-body strength	1-RM bench press (kg)
Power	Counter movement jump (in W or cm) Squat jump (in W or cm) Wingate peak power (W or W⋅kg^−1^)
Hypertrophy	Cross-sectional area of the upper leg muscles (cm) Muscle thickness of upper leg muscles (cm)
Maximal oxygen consumption	$\dot{V}$O_2max_ (mL⋅kg^−1^⋅min^−1^ or mL⋅min^−1^)

*1-RM* one-repetition maximum, $\dot{V}$*O*_*2max*_ maximal oxygen consumption

### Classification of Training Status

To classify training status within concurrent training studies, it is important to note that the training status of participants is not one-dimensional, but can be interpreted both in terms of strength and endurance training status. Unified classification systems have been introduced to categorize participants’ strength training status [27] and endurance training status [28, 29] based on multiple training and physiological performance indicators, such as training experience, attained $\dot{V}$O_2max_, and 1-RM. Training status is typically divided into three categories corresponding to untrained, trained, and highly trained athletes [30, 31]. In this meta-analysis, participants were characterized according to their combination of strength and endurance training status. Strength training status [27] was grouped into untrained, trained (i.e., intermediately trained athletes), and highly trained athletes (i.e., advanced and highly advanced athletes), and endurance training status [28, 29] into untrained, trained (i.e., recreationally trained and trained individuals), and highly trained athletes (i.e., well-trained and professional athletes). The classification framework to determine strength and endurance training status of participants is presented in Table 2 (adapted from [27–29]). To obtain the training status classification, scores were derived for each physiological performance, training, or technique indicator (if present) and were subsequently averaged and rounded. For strength training status, all strength performance indicators were grouped and counted as one score [27]. For endurance training status, the relative values of $\dot{V}$O_2max_ and peak power (compared to body mass) were used by default and absolute values were only scored if relative values were not present. Using this unified classification system, participant groups can be classified for both their strength and endurance training status in order to determine how effects of concurrent training are dependent on training status.

**Table 2:**
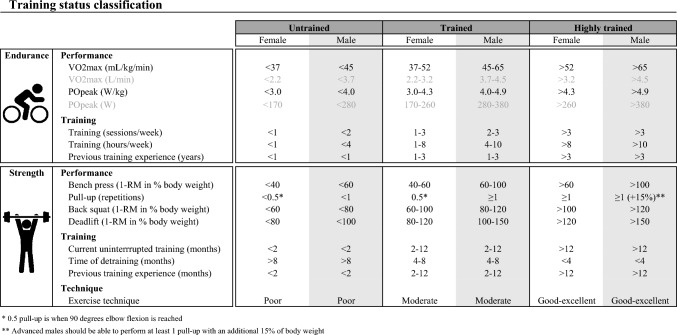
Classification framework to determine strength and endurance training status of male and female participants. Guidelines are adapted from [27–29] and a full explanation of how training status is derived is provided in the description below

As an example of strength training status, a female individual is performing uninterrupted strength training for 6 months (score 2) with no break in her training routine (score 3) and therefore total training experience is also 6 months (score 2). She lifted < 40% of her body mass in the bench press and squatted 80% of her body mass in the back squat (average score 1.5), both with a good exercise technique (score 3). In this case, the average score is 2.3, meaning the individual is considered trained for her strength training status. The values for the deadlift and pull-up are not present and therefore not included in the calculation. Another example for endurance training status concerns a young male individual with a $\dot{V}$O_2max_) of 30 mL/kg/min (score 1) and peak power of 240 W and 2.8 W/kg (score 1; as relative peak power is used by default), who has been training for 6 months (score 1) with an average of 2 h per week (score 1) and 2 sessions per week (score 2). In this case, the average score is 1.2, meaning the individual is considered untrained for his endurance training status. *1-RM* one repetition maximum

### Data Extraction

Two reviewers independently evaluated the full-text articles using a standardized predefined form (RH and SZ). Articles were examined by a third reviewer if consensus was not reached (RW). Data extraction was performed by two reviewers (RH and SZ) and the following data were extracted from the articles: author(s), year of publication, age, sex, sample size, training status (for strength and endurance), training intervention (frequency, duration, intensity, modality), and outcome measures (maximal lower-body and upper-body strength, power, muscle hypertrophy, and $\dot{V}$O_2max_) before and after the intervention. If data on group mean and standard deviation were not presented in the text or tables, then baseline and post-intervention data were requested from the corresponding authors or obtained from figures (if present) using WebPlotDigitizer version 4.5 (Pacifica, CA, USA; [32]). If a particular study included more than one concurrent training group and if these groups were compared to the same control group, results for the intervention groups were combined based on guidelines from the Cochrane handbook [33], to avoid ‘double counts’ of participants in the control group.

### Assessment of Methodological Quality

Methodological quality of identified studies was assessed independently by two reviewers (RH and RW) using the Cochrane risk-of-bias evaluation [33, 34], and consensus was reached after consultation. Reviewers assessed all studies for the risk of selection bias (sequence generation, allocation concealment), detection bias (blinding of outcome assessment), attrition bias (incomplete outcome data), reporting bias (selective reporting), and other biases. Biases were classified for each item as low, high, or unclear risk (i.e., when the risk is unknown or insufficient detail is reported). Relevant biases were assessed one by one according to the recommendation of the Cochrane Bias Methods Group and Statistical Methods Group [33].

### Data Synthesis, Meta-Analysis, and Statistical Analyses

In this study, we compared the effects of (1) concurrent training versus endurance training only (for $\dot{V}$O_2max_) and (2) concurrent training versus strength training only (for other outcomes). Results for maximal strength were subdivided into lower-body and upper-body strength. In accordance with Cochrane recommendations [33], if studies presented multiple methods for the same outcome (e.g. a jump test and Wingate test for power), only one of these was included in the analysis according to the hierarchy presented in Table 1. This was to avoid inclusion of intervention effects that were statistically dependent in the analysis, as these were calculated from the same participants. For example, the hierarchy dictates evaluation of lower-body strength using 1-RM maximal strength during leg press (which is technically easier to execute) over the squat and evaluation of power using countermovement or squat jumps (which are more readily available) over Wingate peak power. Studies or intervention groups were excluded from the analysis if significant baseline differences were observed for the specific outcome measure between the concurrent training and control group or if only percentage changes were reported.

For the meta-analysis, effect sizes of each study were calculated based on standardized mean differences (SMDs) according to the Hedges’ *g* formula using the pooled standard deviation and incorporates an adjustment for small sample bias [35]. A random-effect model [36] was adopted and presented in forest plots, and records were weighted according to the inverse variance method for each outcome measure. Overall effects were evaluated using *Z*-tests and pooled effect sizes were presented with their 95% confidence interval (CI). Heterogeneity was calculated using the *Q*-test and expressed in terms of Chi^2^ and the *I*^2^ statistic. *I*^2^ describes the percentage of the variability that is attributable to heterogeneity rather than chance [37]. Heterogeneity was evaluated based on *I*^2^, with values of 25%, 50%, and 75% representing low, moderate, or high levels of heterogeneity, respectively [38]. Funnel plots with a 95% interval range were made in R for each outcome to quantify potential publication bias. Subgroup analyses were performed to compare concurrent training effects between (1) male and female participants; (2) untrained, trained, and highly trained levels of strength training status; and (3) untrained, trained, and highly trained levels of endurance training status. Last, sensitivity analyses were performed if one or more studies was considered to be an outlier and/or highly influential based on studentized residuals and Cook’s distances [39]. The meta-analysis was conducted using Review Manager (RevMan) software (Version 5.4.1, The Cochrane Collaboration, 2020) and R (Version 4.2.2, 2022) and the code is available in a GitHub repository (https://github.com/StephanvdZwaard/Concurrent-training-review). A significance level α of 0.05 was used to determine statistical differences.

## Results

### Study Characteristics

**Fig. 2:**
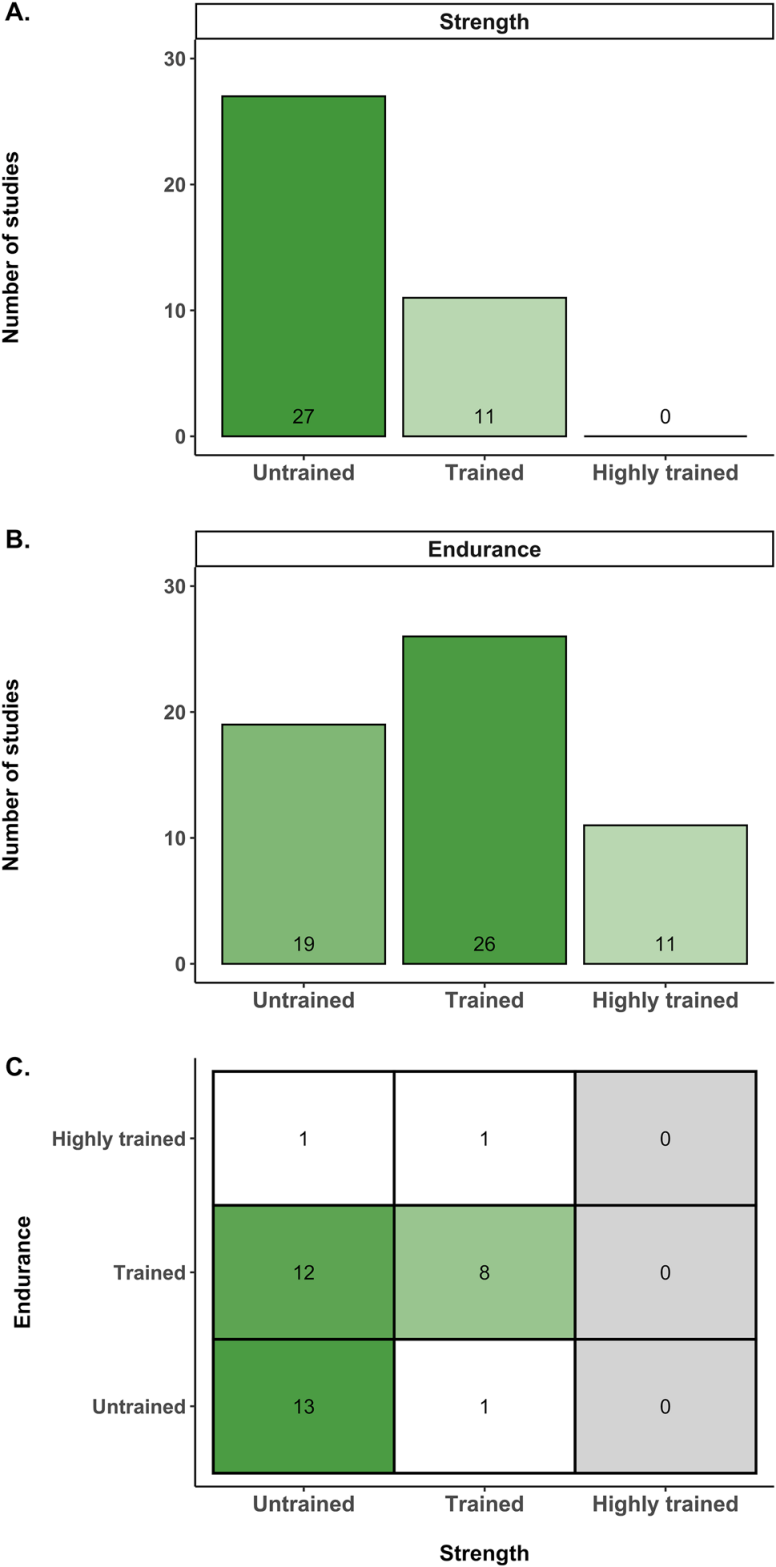
Overview of the training status of the participants in the included studies. Number of studies are reported in which levels of training status could be assessed for (**A**) strength, (**B**) endurance, and (**C**) both strength and endurance. Quantification of training status was performed according to the guidelines in Table 2

The database search resulted in a total of 24,314 articles. After automatic removal of the duplicates, removal of review articles, and screening of the remaining titles and abstracts, 141 articles remained. From these articles, 74 studies [6, 30, 40–111] met the inclusion criteria (see Fig. 1) of which 59 provided the necessary data to be included in the meta-analysis (see Tables S1–3 of the Electronic Supplementary Material [ESM]). Table S4 of the ESM summarizes all relevant characteristics of the studies, participants, and training interventions. The meta-analysis included 1346 participants, of whom 679 performed concurrent training, 270 performed strength training only, and 397 performed endurance training only. Training status of the participants (using Table 2) was determined for strength training status (38 studies), endurance training status (56 studies), and both strength and endurance training status (36 studies). Figure 2 displays the training status level of the participants within these studies, reflecting mostly untrained participants for strength (71%) and predominantly untrained or trained participants for endurance (34% and 46%, respectively). It should be noted that strength as well as endurance training status could only be determined in 61% of the studies included in the meta-analysis. From these studies, only two studies (5%) included participants with highly trained endurance status and no study included highly trained strength participants.

### Sensitivity Analyses

Examination of the studentized residuals and Cook’s distance showed no indications of outliers or overly influential studies in all of the subgroup analyses.

### Risk of Bias Analyses

The overall assessment for the risk of bias is presented in Fig. S17 of the ESM. For the selection bias, a random sequence generation had a low risk in ~ 75% of the studies and allocation concealment was mostly unclear. Detection bias was largely unclear (~ 95%). Attrition bias was low in ~ 80% and high in ~ 20% of the included studies. However, the bias for selective reporting was low in most studies (~ 95%).

### Concurrent Training Effects in Males and Females

**Fig. 3:**
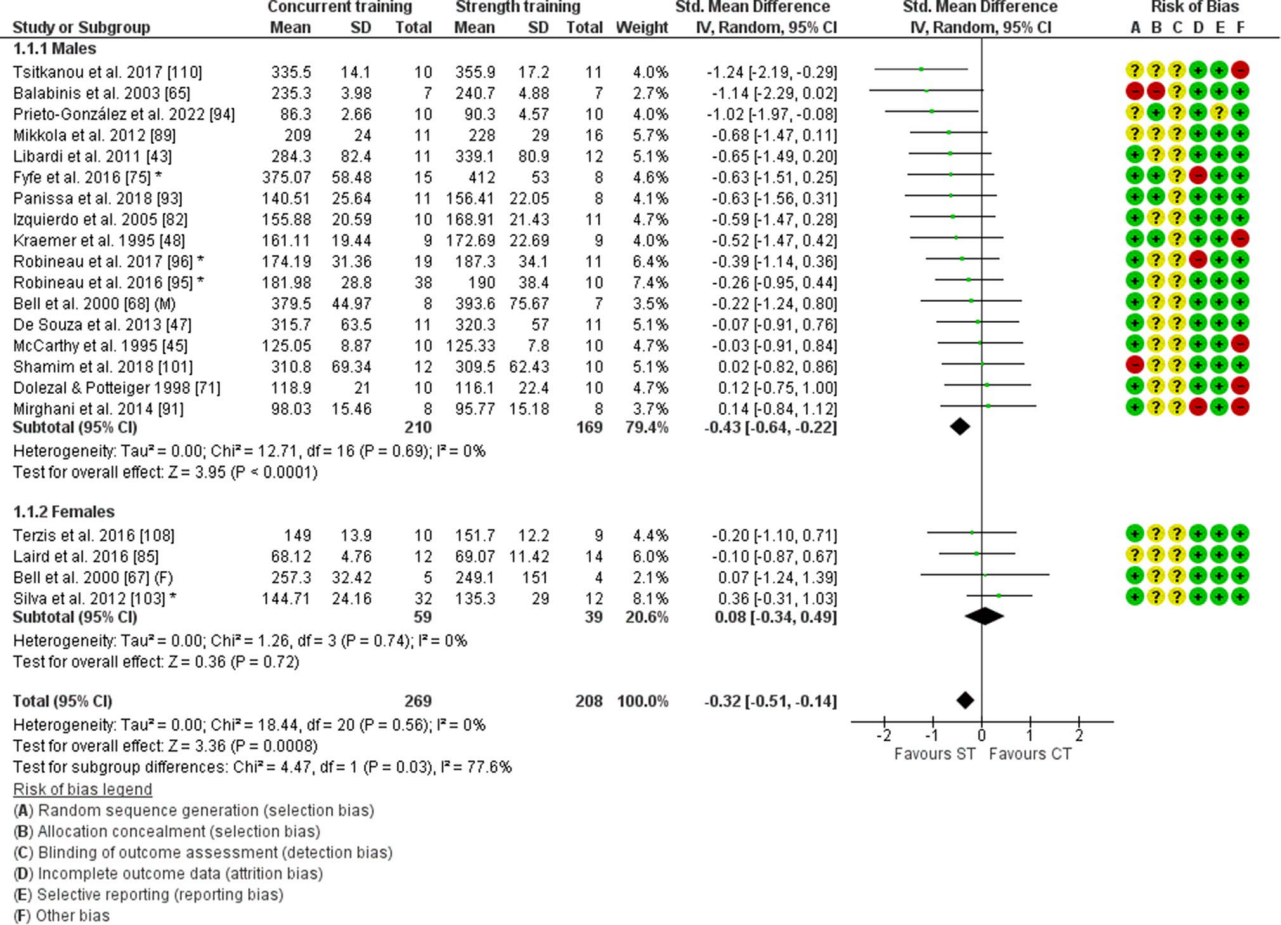
Forest plot of studies comparing differences in adaptations in lower-body strength with concurrent training between males and females. Risk of bias elements are highlighted in the legend below the forest plot and bias was indicated as low (green), unclear (yellow), or high (red). * reflects studies with multiple concurrent training groups that were combined. *CI* confidence interval, *CT* concurrent training group, *SD* standard deviation, *ST* strength training-only control group, *Std.* standard

**Fig. 4:**
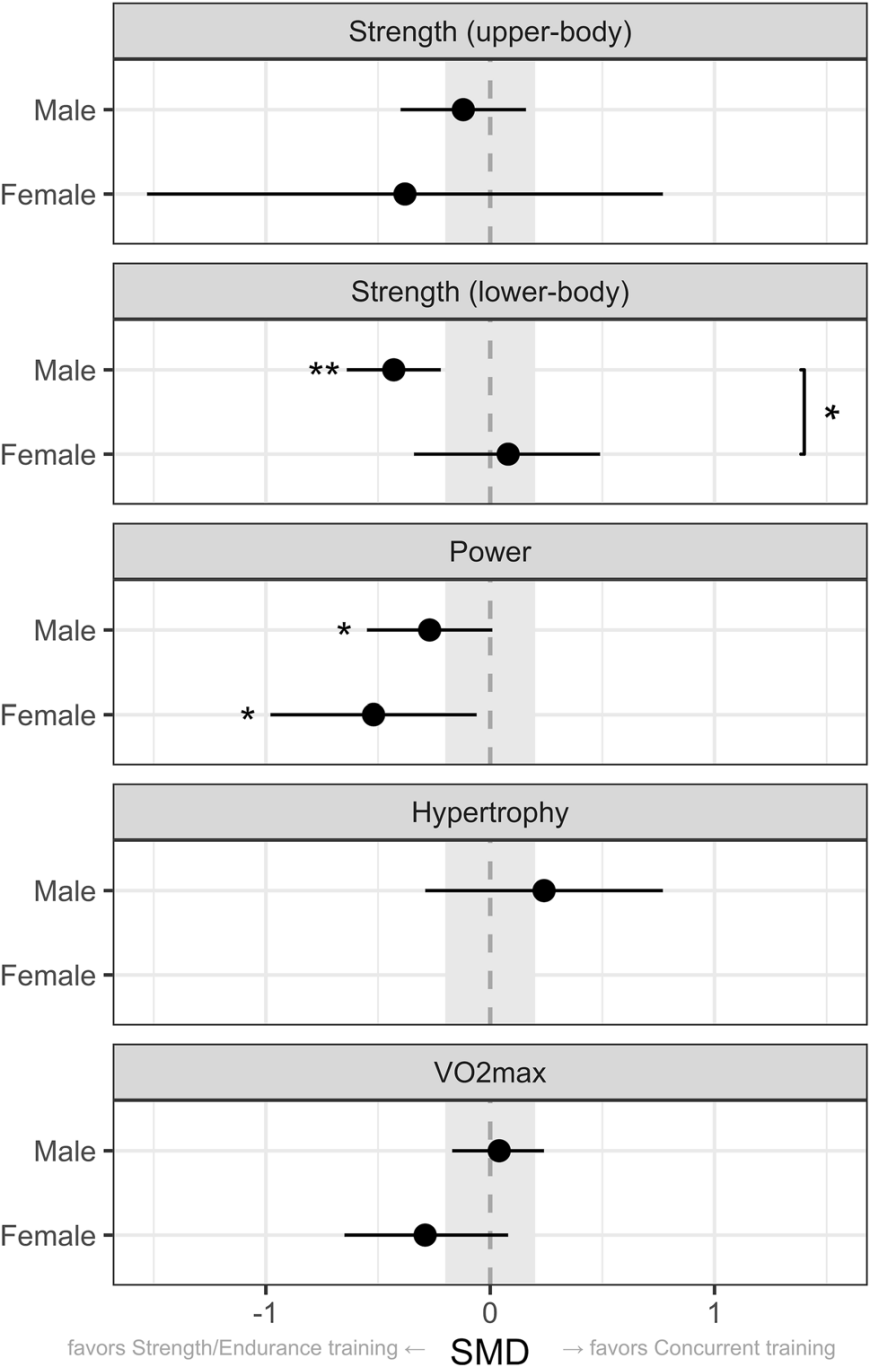
Point range plot demonstrating differences in the effects of concurrent training between males and females for each outcome measure. Effects are reported as the average standardized mean difference (SMD) and their 95% confidence intervals. The gray area highlights the smallest worthwhile change (SMD = 0.2), illustrating what effects can be considered to be non-trivial. Significant effects of concurrent training versus control are reported for males and females separately (next to point range). Significant sex differences were also reported (on the right). *P < 0.05. *CI* confidence interval; *SMD* standardized mean differences

The analysis of lower-body strength included 20 studies [43, 45, 47, 48, 65, 68, 71, 75, 82, 85, 89, 91, 93–96, 101, 103, 108, 110], with 269 participants that performed concurrent training and 208 participants that performed strength training only. Males demonstrated blunted lower-body strength adaptations with concurrent training compared with strength training (− 0.43; 95% CI [− 0.64 to − 0.22], *P* < 0.001), whereas females did not (0.08, 95% CI [− 0.34 to 0.49], *P* = 0.72). These sex differences were significant (*P* = 0.03, Figs. 3 and 4). There was no significant heterogeneity within males (*I*^2^ = 0%, *P* = 0.69) or females (*I*^2^ = 0%, *P* = 0.74). These results highlight a small interference effect for lower-body strength with concurrent training in males, but not in females.

Concurrent training resulted in impaired power gains compared with strength training only in both males (− 0.27, 95% CI [− 0.55 to 0.01], *P* = 0.05) and females (− 0.52, 95% CI [− 0.98 to − 0.06], *P* = 0.03), but without significant sex-differences (*P* = 0.37, based on 13 studies). No sex differences were observed for upper-body strength (*P* = 0.67, based on 13 studies) or $\dot{V}$O_2max_ (*P* = 0.13, based on 32 studies). Data on muscle hypertrophy were available only in males, precluding us to draw any conclusions on sex differences. Forest plots for each outcome measure are also displayed in Figs. S1–5 of the ESM. Our results indicate a small interference with concurrent training for lower-body strength in males, but not in females, and a small-to-moderate interference for power in both sexes.

### Training Status and Interference of Concurrent Training

**Fig. 5:**
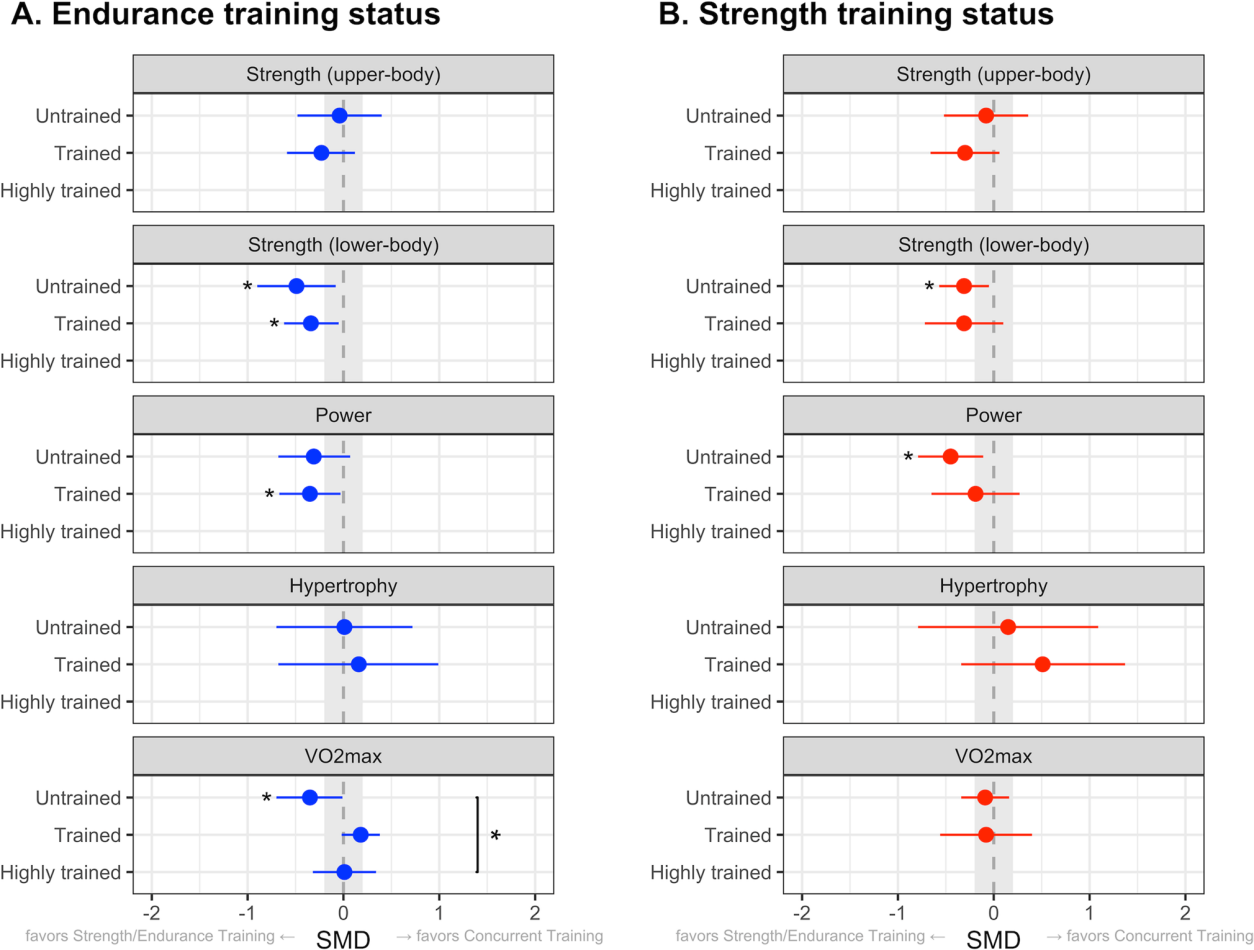
Point range plot demonstrating differences in the effects of concurrent training between levels of training status for endurance (**A**) and strength (**B**) for each outcome measure. Effects are reported as the average standardized mean differences (SMDs) and their 95% confidence intervals. The gray area highlights the smallest worthwhile change (SMD = 0.2), illustrating what effects can be considered non-trivial. Significant effects of concurrent training versus control are reported for each level of training status (next to point range). Significant differences were also reported between training status levels (on the right). *P < 0.05. *CI* confidence interval; *SMD* standardized mean differences

**Fig. 6:**
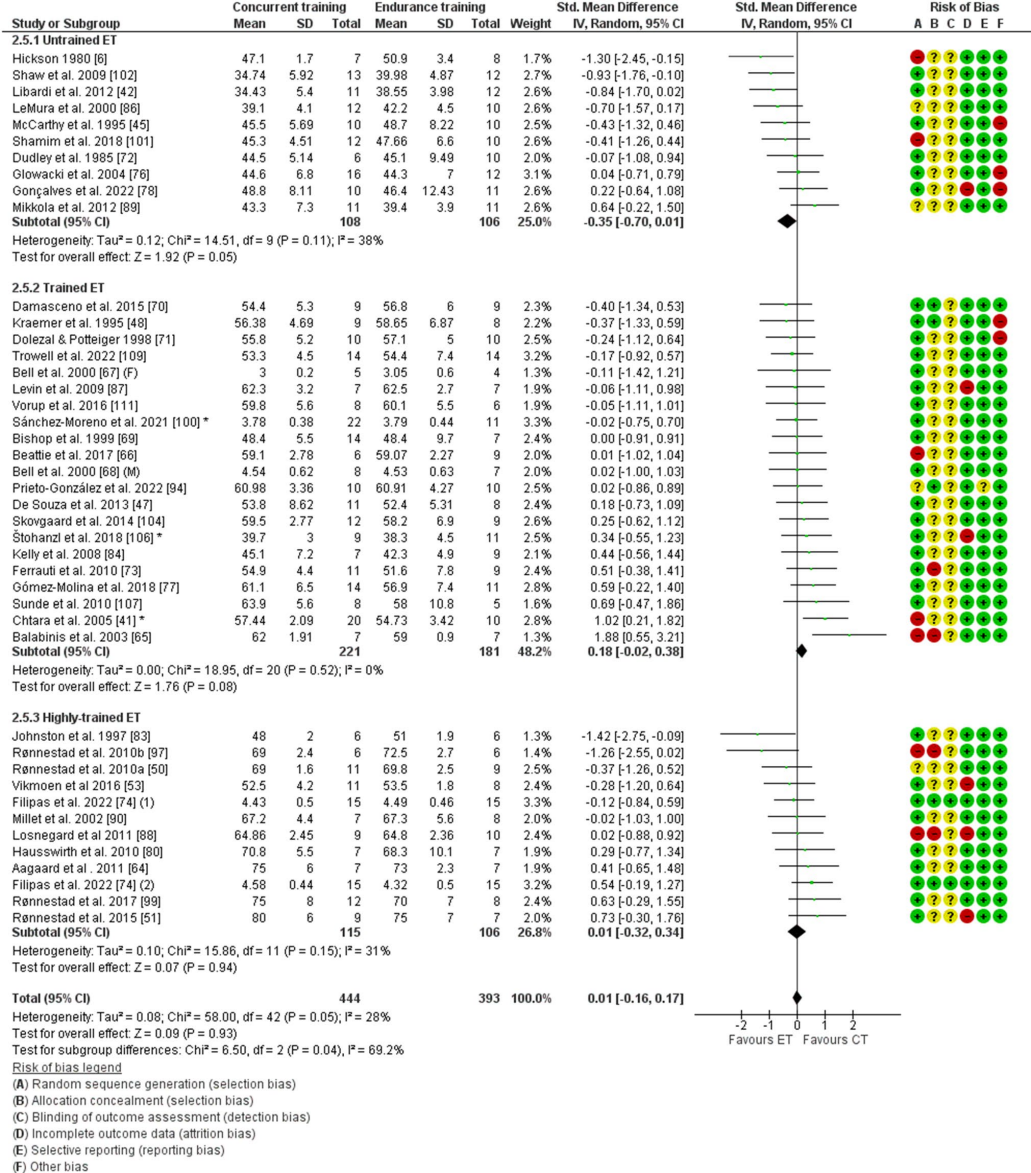
Forest plot of studies comparing differences in adaptations in maximal oxygen consumption with concurrent training between untrained, trained, and highly trained endurance athletes. Risk of bias elements are highlighted in the legend below the forest plot and bias was indicated as low (green), unclear (yellow), or high (red). * reflects studies with multiple concurrent training groups that were combined. *CI* confidence interval, *CT* concurrent training group, *ET* endurance training-only control group, *SD* standard deviation, *Std.* standard

Figure 5 displays the effects of concurrent training related to the participants’ strength and endurance training status. Regarding strength training status, concurrent training resulted in similar adaptations for lower-body strength (*P* = 1.00, based on 16 studies), upper-body strength (*P* = 0.45, based on 14 studies), power (*P* = 0.38, based on 11 studies), and $\dot{V}$O_2max_ (*P* = 0.96, based on 24 studies) between untrained and trained individuals. Similarly, for endurance training status, untrained and trained participants did not show different adaptations for lower-body strength (*P* = 0.55, based on 20 studies), upper-body strength (*P* = 0.50, based on 13 studies), and power (*P* = 0.86, based on 13 studies) with concurrent training. However, improvements in $\dot{V}$O_2max_ with concurrent training were different for untrained, trained, and highly trained endurance athletes (*P* = 0.04, based on 42 studies, see Fig. 6). More specifically, adaptations in $\dot{V}$O_2max_ were slightly blunted in untrained (− 0.35, CI_95%_ [− 0.70 to − 0.01], *P* = 0.05) but not in trained (0.18, 95% CI [− 0.02 to 0.38], *P* = 0.08) and highly trained endurance athletes (0.01, 95% CI [− 0.32 to 0.34], *P* = 0.94). Only data from four studies were available on muscle hypertrophy, which precluded us to perform meaningful subgroup analyses. Limited data other than $\dot{V}$O_2max_ in highly trained endurance athletes precluded us to draw any conclusions for other outcome measures in highly trained athletes.

Lower-body strength adaptations were blunted with concurrent training in untrained and trained endurance athletes (*P* = 0.02 and *P* = 0.02), and untrained strength athletes (*P* = 0.02), but did not reach significance in trained strength athletes (*P* = 0.14). For gains in power, a small-to-moderate interference effect was observed with concurrent training in untrained strength and trained endurance athletes (*P* = 0.01 and *P* = 0.03), whereas trained strength athletes did not demonstrate impaired adaptations in power with concurrent training (*P* = 0.42; Figs. S6–15 of the ESM).

## Discussion

The aim of this meta-analysis was to identify how sex or individual training status affect the adaptations in strength, power, muscle hypertrophy, and $\dot{V}$O_2max_ with concurrent training in healthy adults. Concurrent training resulted in blunted lower-body strength adaptations in males, but not in females. No sex differences were observed for adaptations in upper-body strength, power, or $\dot{V}$O_2max_. There were insufficient studies to investigate muscle hypertrophy. We observed a blunted improvement in $\dot{V}$O_2max_ in untrained, but not in trained or highly trained endurance athletes, and noted a small-to-moderate interference with concurrent training for lower-body strength and power gains in untrained, whereas this did not reach statistical significance in strength-trained athletes. With respect to strength and endurance training status, there were no differences in improvements in maximal lower- and upper-body strength and power between untrained and trained participants.

### Sex Differences in Adaptations to Concurrent Training

Our meta-analysis shows that adaptations to concurrent training differ between sexes. We observed an impaired improvement in maximal lower-body strength in males (SMD: − 0.43), but not in females. There were no significant sex differences for improvements in power, upper-body strength, and $\dot{V}$O_2max_ after concurrent training. When including both males and females, there was a small interference effect for power (SMD: − 0.35) but not for upper-body strength and $\dot{V}$O_2max_. This is in line with two recent meta-analyses that showed that concurrent training hampered improvements in power (SMD: − 0.28), but not in upper-body strength or $\dot{V}$O_2max_, when both sexes are combined [7, 8].

The underlying mechanism for this sex-specific interference remains unknown, but sex differences in muscle and training physiology likely contribute. While sex-related neuromuscular differences could contribute, no sex differences have been observed in the number of motor units or motor unit activation patterns for upper- and lower-body muscles [112, 113], nor in neuromuscular adaptations following exercise training [114].

On average, males tend to have larger muscle fibers and a larger percentage of their cross-sectional area occupied by type II fibers [13, 112, 113, 115, 116]. With these larger fibers, males produce higher absolute forces than females [112]. For strength training adaptations, however, relative improvements in lower-body strength and hypertrophy are similar between males and females [17, 113, 117–119]. Men show larger absolute gains in fiber size [120, 121], which may relate to the observation that glycolytic type II fibers have the larger potential to hypertrophy than type I fibers [122].

Endocrine differences between males and females may contribute to the muscular adaptations with strength training. Circulating testosterone levels in males acutely increase following strength exercise [123, 124], which activates muscle protein synthesis via the anabolic Akt/mTOR pathway and androgen receptors [125, 126]. Despite the lower testosterone levels in females [127], strength exercise-induced muscle protein synthesis seems to be similar between sexes [128–130], suggesting that other factors, such as mechanical signals contribute to the activation of muscle protein synthesis.

The impaired lower-body strength gains in males could also relate to skeletal muscle metabolism and fatigability [131–134], associated with their lower percentage type I fibers. Following concurrent training, this difference could translate into larger residual fatigue from endurance training in men, which may compromise the quality of strength training sessions and subsequent strength gains [135]. Men potentially experience a larger interference effect when combining strength and endurance training because of the tight negative correlation between muscle fiber size and oxidative capacity within skeletal muscle [3, 4] and their larger fiber size. As such, sex-related differences in fiber size, endocrine physiology, and muscle fatigability could help to explain why we observed blunted lower-body strength adaptations in males, but not in females.

### Current Training Status and Interference with Concurrent Training

The adaptations with concurrent training compared to single-mode training may be compromised more in highly trained athletes compared with trained and untrained individuals (known as the ceiling effect) [23]. In contrast, we observed the opposite for adaptations in $\dot{V}$O_2max_ in this meta-analysis, demonstrating blunted adaptations in untrained (SMD: − 0.35), but not in trained and highly trained endurance athletes.

It should be noted that the participant training status classification framework depends on selection criteria for what is regarded ‘untrained,’ ‘trained,’ and ‘highly trained’. A recent meta-analysis [7] distinguished between untrained and trained based on the subgroup description in prior studies (e.g., sedentary, recreationally active). Another meta-analysis [10] distinguished training status based on the World Health Organization physical activity guidelines. Recently, a new classification framework was proposed for determining training status in sport science studies [136]. This framework discriminates very well between world-class, elite, highly trained athletes, and other lower levels of training status, which is very important for avoiding the misuse of the term elite or world-class when addressing participant groups. However, to discriminate between untrained, trained, and highly trained athletes, this framework uses physical activity guidelines and competitive performance characteristics, which are often not reported in the included studies. Based on such criteria, a participant could have a very low endurance capacity ($\dot{V}$O_2max_ of 30 mL/kg/min), but also perform individual competitive exercise, which classify this individual as a “trained participant”, despite being physically unfit. Additionally, such training status classification does not distinguish between endurance and strength-related training status.

Because of these considerations, we decided to apply a training classification framework that (1) is based on physical capacities reflecting the physiology of participants, (2) is based on variables that are commonly reported in previous studies, and (3) that clearly distinguishes between training status in terms of strength and endurance capabilities, which is a necessity for the present meta-analysis. Cut-off values used to distinguish untrained, trained, and highly trained athletes were based on previous classification frameworks that were established based on measurement data from 130 studies in males [28] and 82 studies in females [29] for endurance training status. Although these cut-off points for $\dot{V}$O_2max_ (< 45 mL/kg/min for untrained and > 65 mL/kg/min for highly trained males) may seem like quite the range, we have previously shown that $\dot{{V}}$O_2max_ can range from ~ 25 to 80 mL/kg/min in healthy adults [137]. Therefore, this large range indeed seems to give a broad indication of untrained, trained, or highly trained individuals. The same goes for strength measurements. Therefore, we are confident that our classification framework based on the physical strength and endurance capacities of the participant gives an accurate representation of the untrained, trained, and highly trained athletes.

Untrained participants tend to have less type I fibers with fewer mitochondria and capillaries, a lower stroke volume and cardiac output, and a lower $\dot{V}$O_2max_ compared with endurance-trained athletes [5, 19, 138–141]. Whole-body $\dot{V}$O_2max_ is considered to be limited by oxygen supply to the mitochondria, although $\dot{V}$O_2max_ is well aligned with a small overcapacity of mitochondrial oxidative phosphorylation [137]. Therefore, improvements in $\dot{V}$O_2max_ with training are expected to be accompanied by improvements in both oxygen supply to the mitochondria and mitochondrial oxidative capacity. In response to endurance training, increases in $\dot{V}$O_2max_ are mostly explained by improvements in cardiac output [142–144], although early increases in $\dot{V}$O_2max_ seem to relate to greater maximal arterial-venous oxygen differences [142]. This is in line with the time courses of adaptations to endurance training, showing very rapid increases in mitochondrial oxidative capacity [139, 145, 146] and improvements in capillarization [139, 147] and blood volume [148] with ~ 2 to 4 weeks of training, while central cardiovascular adaptations in maximal stroke volume and cardiac output occur only after month(s) of training [142, 145, 149, 150].

Differences in $\dot{V}$O_2max_ between untrained and trained endurance athletes are largely explained by differences in cardiac output, stroke volume, and mitochondrial oxidative capacity [19, 137]. With concurrent training, untrained individuals are expected to elicit generic adaptations to both strength and endurance training with additive effects [23], that is, untrained individuals may even increase their muscle size after endurance training [151] or their oxidative capacity after strength training [152]. Together with the greater muscle hypertrophy in untrained participants after strength training [153], one may expect untrained participants to show larger increases in the fiber cross-sectional area following concurrent training. As muscle fiber size inverse relates to oxidative capacity [3, 4], this may explain why untrained individuals have more compromised $\dot{V}$O_2max_ adaptations when combining strength and endurance training compared with trained or highly trained endurance athletes.

For maximal lower-body and upper-body strength and power, no differences in adaptations to concurrent training were observed between untrained and trained participants. Not enough studies have been performed to draw conclusions about highly trained athletes. Prior observations in highly trained powerlifters revealed that stronger males, but not females, gain less strength over time, indicative of a ceiling effect [154]. During the general and competitive preparation phase, elite female rowers showed large gains in their muscle physiological cross-sectional area and/or fascicle length [155], indicating that such a ceiling effect was absent during this phase of the season in females. In brief, more studies on highly trained (female) athletes are necessary to evaluate potential interference effects with concurrent training and its underlying mechanisms. Similar to our findings, the meta-analysis of Schumann et al. [7] reported no differences in adaptations with concurrent training between untrained and trained participants for maximal strength, power, and muscle hypertrophy. In contrast, the meta-analysis of Petré et al. [10] observed that maximal lower-body strength gains after concurrent training were impaired in trained, but not in untrained participants. However, they included 27 studies in the meta-analysis (vs 59 studies in our study) and applied a different classification for training status (i.e., several participant groups that were considered moderately trained in [10] were classified as untrained in the our study). The present meta-analysis provides a new perspective on the influence of training status by assessing training status both in terms of strength and endurance capacities of the participants.

### Future Perspectives and Limitations

Perspectives and limitations are considered first regarding sex differences and second related to the influence of training status for adaptations with concurrent training. In general, female participation in research tends to be under-represented compared with male participants in sport science and sports medicine studies (~ 70% of studies included only male participants, often college students [156, 157]). Similarly, we observed more concurrent studies with male participants compared with female participants in this meta-analysis, and our sex-specific conclusions are therefore based on fewer studies. Only four studies on concurrent training effects for muscle hypertrophy were included, all in males, which precluded us to draw any conclusions on the influence of sex. In addition, phases of the menstrual cycle as well as the use of the oral conceptive pill could have an influence on training adaptations and exercise metabolism [117, 158–160], which have not always been controlled for or monitored in training studies with females. Last, highly trained endurance female athletes can suffer from female athlete triad or relative energy deficiency in sport, affecting their reproductive endocrinology and training adaptability [161], which might provide an additional complicating factor in understanding the interference effect of concurrent training in female athletes.

Differences in training volume or design of training interventions could also potentially contribute to observed sex differences (see Table S5 of the ESM). Training adaptations are intrinsically heterogenous, even when performed at the same intensity and load, and therefore, such training studies should include a large enough sample size to account for this intrinsic heterogeneity in training adaptations, independent of an interference effect. Future research could focus on studying the gain and interference effects at different training intensities and volumes, taking into account female sex and pre-training status.

Regarding the assessment of risk of bias, the majority of included studies had some (unclear) risk of bias. Part of this may be owing to the fact that blinding of participants/researchers is not always possible with exercise training interventions or because the required information was not reported in the studies. However, our sensitivity analyses did not indicate the presence of outliers or overly influential studies.

To avoid statistical dependency between analyzed data points, we selected only one measurement method per study if multiple methods were reported for an outcome according to the hierarchy in Table 1. Alternatively, a three-level multivariate meta-analysis could be used to assess a third level of variance within studies, which would allow for the analysis of multiple methods per outcome [162]. Such a statistical analysis could provide additional insights into how adaptations with concurrent training may differ between measurement methods, such as between 1-RM squat and 1-RM leg press for lower-body strength. In the present meta-analysis, however, the number of analyzed studies was too small to perform a three-level meta-analysis [163, 164] and could result in biased estimates of the variance and standard error at the third level (i.e., within studies) [163]. Consequently, we performed a traditional two-level meta-analysis based on random effects [165] and selected one measurement method for each outcome instead. For future systematic reviews and meta-analyses that include more concurrent training studies, preferably 50 or more [164], we encourage the use of three-level meta-analyses to also assess how adaptations with concurrent training may differ between measurement methods.

It should be noted that not all studies reported the required descriptors to determine the training status of participants according to the framework in Table 2. Only ~ 60% of the studies reported sufficient information to determine both strength and endurance training status, and therefore not all studies could be included in this meta-analysis. This complicates meta-analyses, and the general applicability of the study findings. Future concurrent training studies should provide detailed descriptions of their participants’ strength and endurance training status, to allow better assessment of potential differences in the interference effect with concurrent training between untrained, trained, and highly trained individuals.

Furthermore, it should be noted that the framework to determine training status did not account for age. However, this effect is likely marginal for this study, as the average age was 27 ± 7 years (see Table S4 of the ESM). Reductions in strength and $\dot{V}$O_2max_ are expected only with advancing age [166–168], but we do not know how our results extrapolate to older age.

It has been suggested that instead of $\dot{V}$O_2max_, other physiological performance-based parameters such as critical power, could be used to classify endurance training status [169, 170]. $\dot{V}$O_2max_ is appropriate to discriminate between individuals that largely differ in their endurance training status, such as untrained, trained, and highly trained endurance athletes. However, a measure like critical power may be more valuable to discriminate between more homogeneous groups of athletes with rather similar training status, such as well-trained amateurs, sub-elite athletes, and elite athletes [170]. Therefore, adding critical power as a criterion to the framework to classify training status could be worthwhile to describe more specific subgroups, such as highly trained athletes.

In terms of practical advice, individuals and coaches who incorporate concurrent strength and endurance training within their training should consider the potential small-to-moderate interference effects resulting in blunted training adaptations. Not only highly trained athletes, but also untrained individuals should be aware of these effects. Male athletes should be extra cautious when aiming to improve lower-body strength, whereas this seems to be less of an issue in female athletes. In addition, contrary to prior beliefs, these blunted adaptations may also occur for endurance-related outcomes (i.e., $\dot{V}$O_2max_). Therefore, when optimizing an individual prescription of concurrent training in athletes, inter-individual differences in sex and training status should be considered when evaluating magnitudes of the observed adaptations. Furthermore, we encouraged scientists to provide a detailed description of their participants’ strength and endurance training status.

## Conclusions

This meta-analysis shows that concurrent strength and endurance training resulted in blunted lower-body strength adaptations in males, but not in females. Concurrent training also resulted in impaired improvements in $\dot{V}$O_2max_ in untrained, but not in trained or highly trained athletes. Our meta-analysis indicated that highly strength- or endurance-trained athletes are under-represented in the concurrent training literature. In summary, inter-individual differences in sex and training status should be considered when optimizing concurrent training prescription in individual athletes.

### Supplementary Information

Below is the link to the electronic supplementary material.

Supplementary file1 (PDF 6176 KB)

## Declarations

### Availability of data and material

The datasets analyzed during the current study are available in a GitHub repository (https://github.com/StephanvdZwaard/Concurrent-training-review).

### Code availability

The code is available in a GitHub repository (https://github.com/StephanvdZwaard/Concurrent-training-review).
