# Supplementary material for: Concurrent Strength and Endurance Training: A Systematic Review and Meta-Analysis on the Impact of Sex and Training Status
Source: Sports Med. 2023 Oct 17;54(2):485–503. doi: 10.1007/s40279-023-01943-9 (PMC10933151; doi:10.1007/s40279-023-01943-9)
Supplement: Supplementary file 1 — Supplementary file1 (PDF 6176 KB) [file 40279_2023_1943_MOESM1_ESM.pdf]

Electronic Supplementary Material

Concurrent strength and endurance training: a systematic review and meta-analysis on the impact of sex and training status

Raven O. Huiberts[1], Rob C.I. Wüst[1] and Stephan van der Zwaard[1,2]

Sports Medicine

Affiliations: [1] Department of Human Movement Sciences, Faculty of Behavioural and Movement Sciences,Vrije Universiteit Amsterdam, Amsterdam Movement Sciences, The Netherlands;
[2] Department of Cardiology, Amsterdam University Medical Center, University of Amsterdam, The Netherlands

Correspondence: Stephan van der Zwaard, PhD
Department of Cardiology,
Amsterdam University Medical Center
Meibergdreef 9, 1105 AZ, Amsterdam, The Netherlands
Email: s.vanderzwaard@amsterdamumc.nl

Conflict of interest: The authors have no conflicts of interest to declare.

Funding: No financial support was received for the research, authorship, and/or publication of this article

Table S1. Included studies where results from the same study groups were published before.

Included study	Study with same group	Reason for inclusion
Chtara et al. 2008 [1]	Chtara et al. 2005 [2]	Same participants but additional outcome: power (jump height)
Libardi et al. 2012 [3]	Libardi et al. 2011 [4]	Same participants but additional outcome: VO_{2max}
McCarthy et al. 2002 [5]	McCarthy et al. 1995 [6]	Same participants but additional outcome: hypertrophy (cross-sectional area)

Table S2. Excluded studies where results from the same study groups were published before.

Excluded study	Study with same group	Reason for exclusion
De Souza et al. 2014 [7]	De Souza et al. 2013 [8]	Same participants and similar outcomes
Kraemer et al. 2004 [9]	Kraemer et al.1995 [10]	Same participants and similar outcomes
Rønnestad et al. 2011 [11]	Rønnestad et al. 2010a [12]	Same participants and similar outcomes
Rønnestad et al. 2016 [13]	Rønnestad et al. 2015 [14]	Same participants in control group
Vikmoen et al. 2016 [15]	Vikmoen et al. 2016 [16]	Same participants and similar outcomes

Table S3. Excluded studies that were eligible for data extraction, but did not present the necessary data for the meta-analysis.

Excluded study	Reason for exclusion
Beattie et al. 2017 [17]	Significant group differences at baseline
Gergley et al. 2009 [18]	Data was presented in percentage change
Gravelle et al. 2000 [19]	Significant group differences at baseline
Jones et al. 2016 [20]	Data was presented in percentage change
Kawano et al. 2009 [21]	Data was presented in percentage change
Lee et al. 2020 [22]	Data was presented in percentage change
Leveritt et al. 2003 [23]	Data was presented for mixed gender (males + females) and training status differed between CT group and controls
Psilander et al. 2015 [24]	Significant group differences at baseline
Støren et al. 2008 [25]	Data was presented for mixed gender (males + females) and training status differed between CT group and controls
Timmins et al. 2020 [26]	Data was presented in percentage change

Table S4. Characteristics of the participants, training and reported outcomes for the studies included in the meta-analysis.

Study	N	Age	Sex	Duration	Training status		Endurance training			Strength training			Outcomes
					ST	ET	Frequency	Intensity	Mode	Frequency	Exercises	Set x Reps (intensity)	
**Aagaard et al. 2011 [27]**	7	19.1	M	16	ND	3	ND (15.5h/week)	ND (intensity was similar between concurrent and endurance training groups)	Cycling	2-3	Isolated knee extension, incline leg press, hamstring curls, calf raises	4 x 5-12 RM	VO2max
**Balabinis et al. 2003 [28]**	7	22.6	M	7	2	2	4	Continuous running at 70% HRmax and 30-500m intervals at >85% HRmax	Running	4	Bench-press, leg press, half squat, lateral pulldowns.<br>In wk 4 and 5 also plyometrics: front cone hops, diagonal cone hops, lateral cone hops, tuck jump with knees up, incline push-up depth jump, and handstand depth jump	3-6 x 3-6 at 75-95% 1RM (30-40 reps at 40-70% 1RM in last 3 weeks).<br>In weeks 4 and 5, also 2 x 15 plyometrics	Strength (lower)<br>Strength (upper)<br>Power<br>VO2max
**Beattie et al. 2017 [29]**	6	38.0	M	20	1	2	ND (continued with their regular endurance program)	ND (because of the extensive longitudinal nature of the study, aerobic endurance training (intensity) was not controlled in either group)	Cycling	2	Squat jump, trap bar deadlift, Romanian deadlift, split squat, core circuit	2-3 x 3-12 heavy maximal strength and 2-3 x 3-8 light to medium effort. Both days focused on explosive movements.	VO2max
**Bell et al. 1997 [30]**	22	22.4	M/F	16	2	2	3	30-40 min continuous training at VT. 5x3 min interval training performed at 90% VO2max with 3 min recovery	Rowing	3	Bilateral incline (45 degree's) leg press, knee extension, knee flexion, bench press, seated row, lat pulldowns, and arm curls	3-6 x 2-10 at 65-85% 1RM	Strength (lower)<br>Strength (upper)
**Bell et al. 2000 [31]**	13	22.3	M/F	12	ND	2	3	30-42 min continuous training at VT. 4-7x3 min interval training performed at 90% VO2max with 3 min recovery	Cycling	3	Double leg press, single leg knee flexion and extension, double leg calf raises, bench press, seated pulldowns, shoulder press, and bicep curls	2-6 x 4-12 at 72-84% 1RM	Strength (lower)<br>VO2max
**Bishop et al. 1999 [32]**	14	18-42	F	12	ND	2	ND (continued with their regular endurance program	ND (continued with their regular endurance program)	Cycling	2	Squat training on a plyopower resistance machine.	3-5 x 2-8 RM	VO2max

							110.2 ± 29.4 km/week)						
**Chtara et al. 2005 [2] & Chtara et al. 2008* [1]**<br>CT1 (ST → ET)	10	21.4	M	12	ND	2	2	5 x 200m at 100% vVO2max<br>active recovery at 60% vVO2max	Running	2	Circuit consisting of abdominal strengthening, hip extension, back extensors, half squats, forward alternated arm flexions, and forward lunges (in period 1 and 2) and drop jumps from a plinth (0.30–0.60 m), hops, hurdles jumps (0.50–0.70 m), single leg hops, single leg bounds, and multi-jump (in period 3 and 4)	4-5 x 5-30 RM	Power<br>VO2max
CT2 (ET → ST)	10	21.4	M	12	ND	2	2	5 x 200m at 100% vVO2max<br>active recovery at 60% vVO2max	Running	2	Circuit consisting of abdominal strengthening, hip extension, back extensors, half squats, forward alternated arm flexions, and forward lunges (in period 1 and 2) and drop jumps from a plinth (0.30–0.60 m), hops, hurdles jumps (0.50–0.70 m), single leg hops, single leg bounds, and multi-jump (in period 3 and 4)	4-5 x 5-32 RM	Power<br>VO2max
**Damasceno et al. 2015 [33]**	9	34.1	M	8	1	2	ND (continued with their regular endurance program 41.3 ± 9.1 km/week)	Continuous running at 50-70% VO2max	Running	2	Half-squat, leg-press, plantar flexion, and knee extension	2-3 x 3-10 RM	VO2max
**De Souza et al. 2013 [8]**	11	22.5	M	8	1	2	2	15-20 x 60s at 80-100% vVO2max with 45-90s recovery	Running	2	Leg-press 45 °, knee extension and knee flexion exercises	3-5 x 6-12 RM	Strength (lower)<br>Hypertrophy<br>VO2max
**Dolezal & Potteiger 1998 [34]**	10	20.1	M	10	2	2	3	25-40 min at 65-85% HRmax	Running	3	Bench press, lat pulldown, shoulder press, bicep curl, triceps pushdown, back squat, leg extension, leg curl, clean pulls, incline dumbbell press, leg press, seated row, and upright row	3 x 4-15 RM	Strength (lower)<br>Strength (upper)<br>VO2max
**Dudley et al. 1985 [35]**	6	22.2	M/F	7	ND	1	3	5 x 5 min intervals to VO2peak with 5 min recovery	Cycling	3	Knee extension on the Cybex 2 isokinetic loading dynamometer	2 x 26-28 contractions at MVC	VO2max

**Ferraut i et al. 2010 [36]**	11	40.0	M/F	8	1	2	ND (4h/week)	Basic endurance training plus one session of 15 km running at 90–95% of their expected marathon velocity	Running	2	Day 1: Leg press, knee extension, knee flexion, hip extension, ankle extension, Day 2: Reverse fly, bench press, lateral flexion, trunk extension, trunk flexion, trunk rotation	4 x 3-5 RM and 3 x 20-25 RM	VO2max
**Filipas et al. 2022 [37]** CT1 (Polarized)	15	34	M	7	ND	3	6	80% below VT1, 6% between VT1-VT2 14% above VT2	Running	1	Warmup with 20 submaximal vertical jumps, 10 submaximal longitudinal jumps and then 60 drop jumps	2 x 10 jumps at 20 cm 2 x 10 jumps at 40 cm 2 x 10 jumps at 60 cm, all at maximal intensity	VO2max
CT2 (Pyramidal)	15	34	M	7	ND	3	6	77% below VT1, 16% between VT1-VT2 7% above VT2	Running	1	Warmup with 20 submaximal vertical jumps, 10 submaximal longitudinal jumps and then 60 drop jumps	2 x 10 jumps at 20 cm 2 x 10 jumps at 40 cm 2 x 10 jumps at 60 cm, all at maximal intensity	VO2max
**Fyfe et al. 2016 [38]** CT1 (HIT)	8	29.6	M	8	1	2	3	5-11 x 2 min intervals at 120-150% LT1 with 1 min of recovery	Cycling	3	Leg press, bench press, seated row, leg extension and leg curl exercises. flat dumbbell press, lat pulldown, dumbbell lunges	3-5 x 4-12 at 65-90% 1RM	Strength (lower) Strength (upper) Power
CT2 (MICT)	7	29.6	M	8	1	1	3	15-33 min continuous cycling at 80-100% LT1	Cycling	3	Leg press, bench press, seated row, leg extension and leg curl exercises. flat dumbbell press, lat pulldown, dumbbell lunges	3-5 x 4-12 at 65-90% 1RM	Strength (lower) Strength (upper) Power
**Glowacki et al. 2004 [39]**	16	22	M	12	1	1	2-3	20-40 minutes at 65-80% HRR	Running	2-3	Leg press, leg curl, standing calf raise, barbell bench press, lateral pull-down, dumbbell military press, barbell curl and abdominal crunches	3 x 6-10 at 75-85% 1RM (excluding warm-up)	Strength (lower) Strength (upper) Power VO2max
**Gómez-Molina et al. 2018 [40]**	14	20.4	M	8	1	2	3	20-45 min at 70-80% HRmax	Running	2	Squat jump, split scissor jump, double leg bound, alternate leg bound, single leg forward hops, depth jump, double leg hurdle jump, single leg hurdle jump	2-6 x 5-10 at maximal explosive effort	VO2max
**Gonçalves et al. 2022 [41]**	10	38.8	M	12	ND	1	3	15-25 min at 50-60% HRR	Running or cycling	3	horizontal leg press, knee extension machine, bench press, seated knee flexion machine, Smith bench press, lat pull-down machine, seated rowing machine, dumbbell shoulder abduction, dumbbell arm curl, pull-down triceps, abdominal crunch, and trunk extension machine	1-3 x 4-12 RM	VO2max

**Häkkinen et al. 2003 [42]**	11	38	M	21	1	ND	2	30-150 min mainly under ~VT1 and 0-2 x 10 min between ~VT1-VT2 and 0-2 x 5 min above ~VT2	Running or cycling	2	Bilateral and/or unilateral knee extension, bench press, triceps pushdown lateral pull-down, sit up, exercise for the trunk extensors, bilateral/unilateral elbow or knee flexion exercise and leg adduction/abduction exercise	3-6 x 3-15 at 50-80% 1RM	Hypertrophy
**Hausswirth et al. 2010 [43]**	7	30.2	M	5	ND	3	ND (11.7h/week)	Mainly <75% VO2max (81% below VT2 and 19% above VT2)	Running, swimming, and cycling (triathletes)	3	Leg extension, leg press, hamstring curl, leg curl, sit-ups, dead lifts	3-5 x 3-5 at >90% 1RM	VO2max
**Hendrickson et al. 2010 [44]**	15	20	F	8	1	2	3	20-30 min at 75-85% HRmax and 400-800-1200-1600m interval runs close to maximal effort with 1:1 recovery.	Running	3	Squat, stiff-leg deadlift, bench press, lat pull-down, upright row, calf exercises, abdominal work, leg press, incline bench press, seated row, shoulder press.	3 x 3-12 RM	Strength (lower)<br>Strength (upper)<br>Power<br>VO2max
**Hickson 1980 [45]**	7	26	M/F	10	1	1	6	6x5 min at ~VO2max (cycling) with 2 min recovery and 30-40 min running as fast as possible.	Running and cycling	5	Squat, knee flexion, knee extension, leg-press, calf raises, deadlifts, sit-ups	3-5 x 5 at >80% 1RM and 3 x 20 at >80% 1RM for calf raises	Strength (lower)<br>Hypertrophy<br>VO2max
**Izquierdo et al. 2005 [46]**	10	41.8	M	16	1	1	1	30-40 min continuous at 70-90% VO2max and using 30s intervals in week 5 (with 30s recovery)	Cycling	1	Bilateral leg press and bilateral knee extension exercises, bench press, chest press, lateral pull-down, shoulder press, abdominal crunch rotary torso, leg curl and adductor-abductor exercises	3-5 x 5-15 at 30-80% 1RM	Strength (lower)<br>Strength (upper)
**Johnston et al. 1997 [47]**	6	30.3	F	10	2	3	4-5	20-30 miles/week running in steady state with a similar weekly frequency, intensity and distance during the intervention	Running	3	Parallel squat, knee flexion, straight-leg heel raises, seated press, rear lat pulldown, hammer curl, weighted sit ups, lunges, knee extensions, bent-leg heel raises, bench press, seated rows, front lat pull-down, and abdominal curl.	2-3 x 6-20 RM	VO2max
**Kelly et al. 2008 [48]**	7	21.0	F	10	1	2	3	Continuous long distance at 130-150bpm<br>Intervals of 3-8min at 150-190 bpm<br>Intervals of 30s-3 min at ~HRmax	Running	3	Squat, calf raises, hip extension, hip flexion, hamstring curl, seated row, bench press, and abdominal exercises	3 x 5 at 60-85+% RM	VO2max
**Kraemer et al. 1995 [10]**<br>CT1 (upper & lower)	9	23.3	M	12	3	2	4	40 min at 80-85% VO2max and<br>200-800 m intervals at 95-100% VO2max	Running	4	Bench press, fly, lat pull down, arm curl, seated row, sit-up, military press, obliques, sit-ups, calf raises, single leg curl, split squat, leg press, deadlift, double knee extension	2-5 x 5-25 RM	Strength (lower)<br>Strength (upper)<br>Power<br>VO2max

CT2 (upper body)	9	22.9	M	12	2	2	4	40 min at 80-85% VO2max and 200-800 m intervals at 95-100% VO2max	Running	4	Bench press, fly, lat pull down, arm curl, seated row, sit-up, military press, obliques, sit-ups	2-5 x 5-25 RM	Strength (lower) Strength (upper) Power VO2max
**Laird et al. 2016 [49]**	12	20.2	F	11	1	1	3	8 x 4 min bouts of 20s at 110-120% vVO2max with 10s recovery	Running	3	Back squat, bent over row, bench press, sit-ups, squat jump, deadlift, standing press, back extension	3-5 x 3-10 at 70-88% 1RM	Strength (lower) Power
**Lemura et al. 2000 [50]**	12	19.0	F	16	ND	1	2	30-45 min at 70-85% HRmax	Running, Rowing or Cycling	2	Leg extension, leg press, leg curl, triceps extension, bicep curl, chest press, decline press, deltoid lateral raises, behind-neck pullover, pectoral adduction and abduction crunches	2-3x8-10 at 60-70% 1RM	VO2max
**Levin et al. 2009 [51]**	7	25	M	6	2	2	ND (8.8h/week)	ND (continued with their regular endurance program)	Cycling	3	Session 1: Jump squats, single-leg jump squat, clean grip deadlift, single-leg calf raises, back extension<br>Session 2: Lunges, squats, straight-leg deadlift, seated calf raises, inclined crunches<br>Session 3: Single-leg leg press, knee extension, knee flexion, standing calf raises, abdominal crunches	3 x 6 RM<br>3 x 12 RM<br>4 x 5 RM	VO2max
**Libardi et al. 2011 [4] & Libardi et al. 2012* [3]**	11	48.5	M	16	1	1	3	30 min at 50-85% VO2max	Running	3	Leg press, leg curl, leg extension, calf raises, bench press, lat pull-down, arm curl.	3 x 8-10 RM	Strength (lower) Strength (upper) VO2max
**Losnegard et al. 2011 [52]**	9	21.2	M/F	12	ND	3	ND (15.2h/week)	ND (continued with their regular endurance program)	Cross country	1-2	Half squat, seated pull-down, standing double-poling and triceps press, and optional lower back and abdominal exercises	warm-up: 3 x 10-6-3 at 40-60-80% 1RM<br>training:3-4 x 4-10 RM	VO2max
**McCarthy et al. 1995 [6] & McCarthy et al. 2002* [5]**	10	27.3	M	10	1	1	3	30-50 min at 70% HRR	Cycling	3	Parallel squat, bench press, standing curl, knee extension, leg curl, wide grip lat pull-down, overhead press, heel raises	warm-up: 1 x 5-7 at 75%RM<br>training: 3 x 5-7 RM	Strength (lower) Strength (upper) Power Hypertrophy VO2max
**Mikkola et al. 2012 [53]**	11	37	M	21	1	1	2	30-90 min below VT1<br>0-2 x 10 min between VT1-VT2<br>0-2 x 5 min > VT2	Cycling or Nordic walking	2	Leg press, knee extension, bench press or lat pull down, triceps pushdown or biceps curl, sit-up exercise or trunk extensors exercise, knee flexion or calf raises, and leg adduction or abduction exercises	2-4 x 3-15 at 50-80% 1RM (leg press and knee extension; with 20% at 50-60% 1RM) and<br>3-5 x 8-15 RM (other exercises)	Strength (lower) VO2max

**Millet et al. 2002 [2002]**	7	24.3	M	14	ND	3	ND (continued with their regular endurance program of 20h/week)	Mainly <70% VO2max	Running, swimming, and cycling (triathletes)	2	Hamstring curl, leg press, seated press, parallel squat, leg extension, and heel raise	3-5 x 3-5 at >90% 1RM	VO2max
**Mirghani et al. 2014 [55]**	8	21.0	M	8	2	ND	2	16-30 min at 65-80% HRmax	Running	2	Bench press, toe raise, shoulder press, squat, lateral pull down and leg curl	2-3 x 6-10 at 55-85% 1RM	Strength (lower)<br>Strength (upper)
**Nelson et al. 1990 [56]**	5	26.0	M	20	1	1	4	30-60 min at 75-85% HRmax	Cycling	4	Knee extension and knee flexion 30°/sec	3 x 6 RM	VO2max
**Panissa et al. 2018 [57]**	11	24.5	M	12	ND	ND	2	Intervals of 1 min at 100% MAV with 1 min recovery until reaching 5 km	Running	2	Bench press, half-squat, triceps extension, leg extension, seated row, leg curl, and arm curl	3 x 8-12 RM	Strength (lower)
**Prieto-González et al. 2022 [58]**	10	34.3	M	12	1	2	1-2	50-60 min running at 117-162bpm (first 6 weeks)<br>10-16 x 1-3 min intervals at 159-180 bpm with 2-8 min recovery	Running	1-2	Squat, leg curl, calf raises, hurdle hops, extended bounds, sprints 100-115% maximal aerobic speed	4-5 x 4-14 at 64-86% 1RM	Strength (lower)<br>Power<br>VO2max
**Robineau et al. 2016 [59]**<br>CT1 (0h recovery)	15	24.3	M	7	1	2	2	3x6 min bouts with 15s intervals at 120% MAV (>90% HRmax) with 15s recovery	Running	2	Half squat, leg press, bench press, bench row, core, hamstrings exercise and plyometric jumps	3-4 x 3-10 at 70-90% 1RM	Strength (lower)<br>Strength (upper)<br>Power
CT2 (6h recovery)	11	28.0	M	7	1	2	2	3x6 min bouts with 15s intervals at 120% MAV (>90% HRmax) with 15s recovery	Running	2	Half squat, leg press, bench press, bench row, core, hamstrings exercise and plyometric jumps	3-4 x 3-10 at 70-90% 1RM	Strength (lower)<br>Strength (upper)<br>Power
CT3 (24h recovery)	12	24.8	M	7	1	2	2	3x6 min bouts with 15s intervals at 120% MAV (>90% HRmax) with 15s recovery	Running	2	Half squat, leg press, bench press, bench row, core, hamstrings exercise and plyometric jumps	3-4 x 3-10 at 70-90% 1RM	Strength (lower)<br>Strength (upper)<br>Power
**Robineau et al. 2017 [60]**<br>CT1 (SIT)	10	26.4	M	8	3	2	2	4-8 x 30s all-out intervals with 4 min recovery	Running	2	Half squat, deadlift, leg extension, bench press, bench row, core, hamstrings exercise, plyometric jumps	3 x 3-10 at 70-90% 1RM	Strength (lower)<br>Strength (upper)<br>Power

CT2 (HIT)	9	25.0	M	8	2	2	2	2 x 8-12 min bouts of 30s intervals at 100% MAV with 30s recovery at 50% MAV	Running	2	Half squat, deadlift, leg extension, bench press, bench row, core, hamstrings exercise, plyometric jumps	3 x 3-10 at 70-90% 1RM	Strength (lower)<br>Strength (upper)<br>Power
**Rønnestad et al. 2010a [12]**	11	27	M/F	12	ND	3	ND (10h/week)	Mainly 60-87% HRmax	Cycling	2	Half squat, one-legged leg press, one legged hip flexion, toe raises	3 x 4-10 RM	VO2max
**Rønnestad et al. 2010b [61]**	6	29	M/F	12	ND	3	ND (11.1h/week)	Mainly 60-87% HRmax	Cycling	2	Half squat, one-legged leg press, one legged hip flexion, toe raises	3 x 4-10 RM	VO2max
**Rønnestad et al. 2015 [14]**	9	19.1	M	25	ND	3	ND (11.3h/week in first 10 weeks; 15.2h/week in final 15 weeks)	Mainly 60-87% HRmax	Cycling	1-2	Half squat, one-legged leg press, one-legged hip flexion, toe raises	3 x 4-10RM (first 12 weeks)<br>3 x 5 at 8-10RM (last 13 weeks)	VO2max
**Rønnestad et al. 2017 [62]**	12	19	M/F	10	ND	3	ND (12.3h/week)	Mainly 60-87% HRmax	Cycling	2	Half squat, one-legged leg press, one legged hip flexion, toe raises	3 x 4-10 RM	VO2max
**Sánchez-Moreno et al. 2021 [63]**<br>CT1 (velocity loss 15%)	11	25	M	8	2	2	2	4-14 x 30s at 80-120% vVO2max with 1-2 min recovery	Running	2	Full squat	3 x 3.2-5.8 at 58-80% 1RM	VO2max
CT2 (velocity loss 45%)	11	25	M	8	2	2	2	4-14 x 30s at 80-120% vVO2max with 1-2 min recovery	Running	2	Full squat	3 x 6-15.5 at 58-80% 1RM	VO2max
**Shamim et al. 2018 [64]**	12	26	M	12	2	1	3	MICT at 50% MAP Intervals at 70% MAP with 60s recovery and intervals at 100% MAP with 20-60 sec recovery at 40% MAP	Cycling	3	Leg press, knee extension, bench press, incline bench, Romanian deadlift, sit-ups, triceps extension, dumbbell row, dumbbell shrugs, seated overhead, biceps curl, hip thrusts, lunges	2-5 x 2-15 at 60-98% 1RM	Strength (lower)<br>Strength (upper)<br>Power<br>Hypertrophy<br>VO2max
**Shaw et al. 2009 [65]**	13	26	M	16	ND	1	3	22 min at 60% HRmax	Running, rowing and cycling	3	Shoulder press, latissimus dorsi pull-downs, seated rows, unilateral leg presses, unilateral knee extensions, unilateral hamstring curls, crunches	2 x 15 at 60% 1RM	VO2max
**Silva et al. 2012 [66]**<br>CT1 (MICT running)	10	22.3	F	11	1	1	2	20-30 min at 95% HR at VT2	Running	2	Inclined leg press, knee extension, leg curl, bench press, inverted fly, upright row, and sit-ups	2-3 x 8-18 RM	Strength (lower)<br>Strength (upper)
CT2 (HIT running)	11	24.3	F	11	1	1	2	1 min at 100% vVO2max with 1 min recovery at 50% vVO2max for 20-30 min	Running	2	Inclined leg press, knee extension, leg curl, bench press, inverted fly, upright row, and sit-ups	2-3 x 8-18 RM	Strength (lower)<br>Strength (upper)

CT3 (MICT cycling)	11	21.8	F	11	1	1	2	20-30 min at 95% HR at VT2	Cycling	2	Inclined leg press, knee extension, leg curl, bench press, inverted fly, upright row, and sit-ups	2-3 x 8-18 RM	Strength (lower)<br>Strength (upper)
**Skovgaard et al. 2014 [67]**	12	31.1	M	8	2	2	4	2 sessions: 4-12 x 30s all-out with 3 min recovery<br>1 session: 4 x 4min with 2 min recovery at >85% HRmax<br>1 session: 40-70 min continuous running at 75-85% HRmax	Running	2	Squat, deadlift, and leg press as exercises.	3-4 x 4-8 at 15-4RM	VO2max
**Spiliopoulou et al. 2021 [68]**	9	21.8	F	6	1	1	3	10 x 1min at 100% MAP with 1 min recovery	Cycling	3	Counter movement jumps, drop jumps, eccentric squat, half squat	4-6 x 2 at 40-65% 1RM (half squats)<br>6-8 x 3 CMJ and drop jumps at maximal effort	Strength (lower)<br>Power
**Štohanzl et al. 2018 [69]**<br>CT1 (30min ST)	11	32	F	10	1	1	ND<br>(2.5h/week)	2.5h < VT2	Running	1	Calf jumps, low skater jumps, push-ups on the bench, half squat jumps, lunges, step-ups on bench, plank	3-4 x 12-30<br>3-4 x 30-40s (plank)	VO2max
CT2 (60min ST)	9	32	F	10	1	2	ND<br>(2h/week)	2h < VT2	Running	2	Calf jumps, low skater jumps, push-ups on the bench, half squat jumps, lunges, step-ups on bench, plank	3-4 x 12-30<br>3-4 x 30-40s (plank)	VO2max
**Sunde et al. 2010 [70]**	8	29.9	M/F	8	ND	2	ND<br>(continued their regular endurance program of ~9h/week)	ND<br>(training intensity zones were monitored for each subject based on 60–85%, 85–90%, and 90–95% of HRmax, but these were not reported)	Cycling	3	Half squats	4 x 4 RM	VO2max
**Terzis et al. 2016 [71]**	10	21.6	F	6	ND	2	3	30 min at 60-70% HRmax	Running	3	Half squats, counter movement jumps, drop jumps	6 x 2 at 40-65% 1RM (half squats)<br>6-8 x 3 CMJ and drop jump at maximal effort	Strength (lower)<br>Power
**Trowell et al. 2022 [72]**	14	33.1	M/F	10	1	2	ND<br>(continued their regular endurance program of 4.5 times/week and 288 min/week)	ND<br>(total training time was matched for both CT and ET groups)	Running	2	Ankle bounce, back squat, hurdle jumps, frontal plank, high knee drill or A-skip drill, single leg deadlift, split squat jump, side-stepping, counter movement jump or drop jump, glute bridge	3-5 x 6-20 at 70% 1RM<br>3-5 x 60s (plank)<br>3-5 x 15 (hurdles)<br>3-5 x 20m (drills)	VO2max

**Tsitkanou et al. 2017 [73]**	10	21.8	M	8	ND	1	2	10 x 60s at 100% MAP with 1 min recovery	Cycling	2	Leg press, half squat<br>Abdominal crunches, lateral crunches, dorsal raises	4 x 6 at 80-100% 6RM and 2 x 10 for crunches and dorsal raises.	Strength (lower)<br>Hypertrophy
**Vikmoen et al. 2016 [16]**	11	31.5	F	11	1	3	4.1	63% below ~VT1<br>21% ~VT1-VT2<br>16% above ~VT2	Cycling	2	Half squat, one-legged leg press, on legged hip flexion, toe raises	3 x 4-10 RM	VO2max
**Vorup et al. 2016 [74]**	9	39.2	M	8	1	2	3.7	2 sessions: 2 x 100m at 75% Vmax + 4-10 x 30s at 90-95% Vmax with 3 min recovery<br>1 session: continuous running at 80% HRmax (2-17km)<br>1 session: 8 x 2 min at >90% HRmax with 1 min recovery	Running	2	Squat, leg press, and deadlift	1-4 x 4-10 RM	VO2max

M= male, F= female, for training status: ST= strength training status, ET = endurance training status, 1= untrained, 2= trained, 3=highly-trained, ND= not determined, no available data, h=hours, *=studies same CT group, bpm= beats per minute; HIT = high intensity interval training; HRmax= maximal heart rate; HRR = heart rate reserve; LT1 = first lactate threshold; MAP= maximal aerobic power; MAV= maximal aerobic velocity; MICT = moderate intensity continuous training; RM = repetition maximum; SIT = sprint interval training; VO2max= maximal oxygen uptake; VT1= first ventilatory threshold; VT2= second ventilatory threshold; vVO2max=velocity at maximal aerobic capacity;

Table S5. Differences in training status and training regimen between males and females for comparisons related to lower-body strength.

Strength (lower-body)	Males		Females	
**N**	210		59	
**Training status**	Strength	Endurance	Strength	Endurance
Untrained	59%	39%	75%	75%
Trained	28%	52%	0%	25%
Highly trained	5%	0%	0%	0%
Not determined	9%	9%	25%	0%
**Training duration**				
<8 weeks	21%		17%	
8-12 weeks	63%		83%	
>12 weeks	15%		0%	
**Training frequency**				
≤4 times / week	61%		54%	
5-6 times / week	31%		46%	
>6 times / week	8%		0%	
**Training mode (ET)**				
Cycling	36%		27%	
Running	64%		73%	
Other	0%		0%	

Study or Subgroup	CT Mean	CT SD	CT Total	ST Mean	ST SD	ST Total	Weight	Std. Mean Difference IV, Random, 95% CI	A	B	C	D	E	F
**1.1.1 Males**														
Tsitkanou et al . 2017	335.5	14.1	10	355.9	17.2	11	4.0%	-1.24 [-2.19, -0.29]	?	?	?	+	+	−
Balabinis et al. 2003	235.3	3.98	7	240.7	4.88	7	2.7%	-1.14 [-2.29, 0.02]	−	−	?	+	+	+
Prieto-González et al. 2022	86.3	2.66	10	90.3	4.57	10	4.0%	-1.02 [-1.97, -0.08]	?	+	?	+	?	+
Mikkola et al. 2012	209	24	11	228	29	16	5.7%	-0.68 [-1.47, 0.11]	?	?	?	+	+	+
Libardi et al. 2011	284.3	82.4	11	339.1	80.9	12	5.1%	-0.65 [-1.49, 0.20]	+	?	?	+	+	+
Fyfe et al. 2016 *	375.07	58.48	15	412	53	8	4.6%	-0.63 [-1.51, 0.25]	+	+	?	−	+	+
Panissa et al. 2018	140.51	25.64	11	156.41	22.05	8	4.1%	-0.63 [-1.56, 0.31]	+	+	?	+	+	+
Izquierdo et al. 2005	155.88	20.59	10	168.91	21.43	11	4.7%	-0.59 [-1.47, 0.28]	+	?	?	+	+	+
Kraemer et al. 1995	161.11	19.44	9	172.69	22.69	9	4.0%	-0.52 [-1.47, 0.42]	+	+	?	+	+	−
Robineau et al. 2017 *	174.19	31.36	19	187.3	34.1	11	6.4%	-0.39 [-1.14, 0.36]	+	?	?	−	+	+
Robineau et al. 2016 *	181.98	28.8	38	190	38.4	10	7.4%	-0.26 [-0.95, 0.44]	+	?	?	+	+	+
Bell et al. 2000 (M)	379.5	44.97	8	393.6	75.67	7	3.5%	-0.22 [-1.24, 0.80]	+	?	?	+	+	+
De Souza et al. 2013	315.7	63.5	11	320.3	57	11	5.1%	-0.07 [-0.91, 0.76]	+	?	?	+	+	+
McCarthy et al. 1995	125.05	8.87	10	125.33	7.8	10	4.7%	-0.03 [-0.91, 0.84]	+	?	?	+	+	−
Shamim et al. 2018	310.8	69.34	12	309.5	62.43	10	5.1%	0.02 [-0.82, 0.86]	−	?	?	+	+	+
Dolezal & Potteiger 1998	118.9	21	10	116.1	22.4	10	4.7%	0.12 [-0.75, 1.00]	+	?	?	+	+	−
Mirghani et al. 2014	98.03	15.46	8	95.77	15.18	8	3.7%	0.14 [-0.84, 1.12]	+	?	?	−	+	−
**Subtotal (95% CI)**			**210**			**169**	**79.4%**	**-0.43 [-0.64, -0.22]**						
Heterogeneity: Tau² = 0.00; Chi² = 12.71, df = 16 (P = 0.69); I² = 0%														
Test for overall effect: Z = 3.95 (P < 0.0001)														
**1.1.2 Females**														
Terzis et al. 2016	149	13.9	10	151.7	12.2	9	4.4%	-0.20 [-1.10, 0.71]	+	?	?	+	+	+
Laird et al. 2016	68.12	4.76	12	69.07	11.42	14	6.0%	-0.10 [-0.87, 0.67]	?	?	?	+	+	+
Bell et al. 2000 (F)	257.3	32.42	5	249.1	151	4	2.1%	0.07 [-1.24, 1.39]	+	?	?	+	+	+
Silva et al. 2012 *	144.71	24.16	32	135.3	29	12	8.1%	0.36 [-0.31, 1.03]	+	?	?	+	+	+
**Subtotal (95% CI)**			**59**			**39**	**20.6%**	**0.08 [-0.34, 0.49]**						
Heterogeneity: Tau² = 0.00; Chi² = 1.26, df = 3 (P = 0.74); I² = 0%														
Test for overall effect: Z = 0.36 (P = 0.72)														
**Total (95% CI)**			**269**			**208**	**100.0%**	**-0.32 [-0.51, -0.14]**						
Heterogeneity: Tau² = 0.00; Chi² = 18.44, df = 20 (P = 0.56); I² = 0%														
Test for overall effect: Z = 3.36 (P = 0.0008)														
Test for subgroup differences: Chi² = 4.47, df = 1 (P = 0.03), I² = 77.6%														

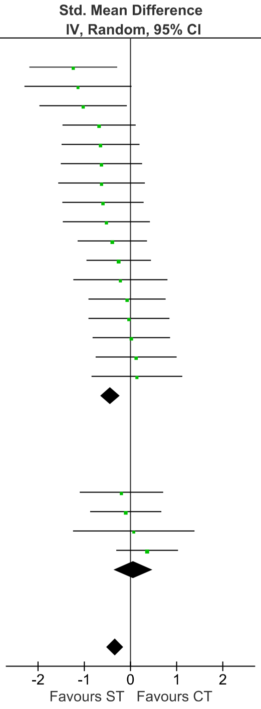

Risk of bias legend
(**A**) Random sequence generation (selection bias)
(**B**) Allocation concealment (selection bias)
(**C**) Blinding of outcome assessment (detection bias)
(**D**) Incomplete outcome data (attrition bias)
(**E**) Selective reporting (reporting bias)
(**F**) Other bias

Figure S1. Forest plot of studies comparing differences in adaptations in lower-body strength with concurrent training between males and females.

Study or Subgroup	CT Mean	CT SD	CT Total	ST Mean	ST SD	ST Total	Weight	Std. Mean Difference IV, Random, 95% CI	A	B	C	D	E	F
**1.2.1 Males**														
Izquierdo et al. 2005	69.75	11.93	10	82.92	7.2	11	6.0%	-1.30 [-2.26, -0.34]	+	?	?	+	+	+
Mirghani et al. 2014	64.85	7.28	8	70.3	8.14	8	5.4%	-0.67 [-1.68, 0.35]	+	?	?	−	+	−
Libardi et al. 2011	84.4	13.5	11	90	14.8	12	7.5%	-0.38 [-1.21, 0.45]	+	?	?	+	+	+
Robineau et al. 2016 *	88.34	19.02	38	95.3	14.7	10	9.6%	-0.38 [-1.08, 0.33]	+	?	?	+	+	+
Robineau et al. 2017 *	97.24	18.75	19	99.5	14.2	11	8.8%	-0.13 [-0.87, 0.62]	+	?	?	−	+	+
Kraemer et al. 1995	95.14	14.86	9	97.03	20.54	9	6.3%	-0.10 [-1.03, 0.82]	+	+	?	+	+	−
Dolezal & Potteiger 1998	92.9	21.5	10	94.3	15.3	10	6.9%	-0.07 [-0.95, 0.81]	+	?	?	+	+	−
Shamim et al. 2018	81.3	20.46	12	82.8	21.81	10	7.4%	-0.07 [-0.91, 0.77]	−	?	?	+	+	+
Balabinis et al. 2003	110.8	3.93	7	110.5	2.14	7	5.2%	0.09 [-0.96, 1.14]	−	−	?	+	+	+
McCarthy et al. 1995	85.73	7.93	10	84.36	6.77	10	6.9%	0.18 [-0.70, 1.06]	+	?	?	+	+	−
Fyfe et al. 2016 *	90	18.96	15	83	22	8	7.0%	0.34 [-0.53, 1.20]	+	+	?	−	+	+
Glowacki et al. 2004	99.5	17.8	16	86.5	18.6	13	8.6%	0.70 [-0.06, 1.45]	+	?	?	+	+	−
**Subtotal (95% CI)**			**165**			**119**	**85.7%**	**-0.12 [-0.40, 0.16]**						
Heterogeneity: Tau² = 0.05; Chi² = 13.95, df = 11 (P = 0.24); I² = 21%														
Test for overall effect: Z = 0.86 (P = 0.39)														
**1.2.2 Females**														
Bell et al. 1997 (F)	46.2	4.5	8	51.5	4.5	5	3.9%	-1.10 [-2.32, 0.13]	−	−	?	−	+	+
Silva et al. 2012 *	35.97	5.06	32	35.4	6.9	12	10.4%	0.10 [-0.56, 0.76]	+	?	?	+	+	+
**Subtotal (95% CI)**			**40**			**17**	**14.3%**	**-0.38 [-1.53, 0.77]**						
Heterogeneity: Tau² = 0.46; Chi² = 2.83, df = 1 (P = 0.09); I² = 65%														
Test for overall effect: Z = 0.65 (P = 0.51)														
**Total (95% CI)**			**205**			**136**	**100.0%**	**-0.14 [-0.40, 0.12]**						
Heterogeneity: Tau² = 0.06; Chi² = 16.80, df = 13 (P = 0.21); I² = 23%														
Test for overall effect: Z = 1.04 (P = 0.30)														
Test for subgroup differences: Chi² = 0.18, df = 1 (P = 0.67), I² = 0%														

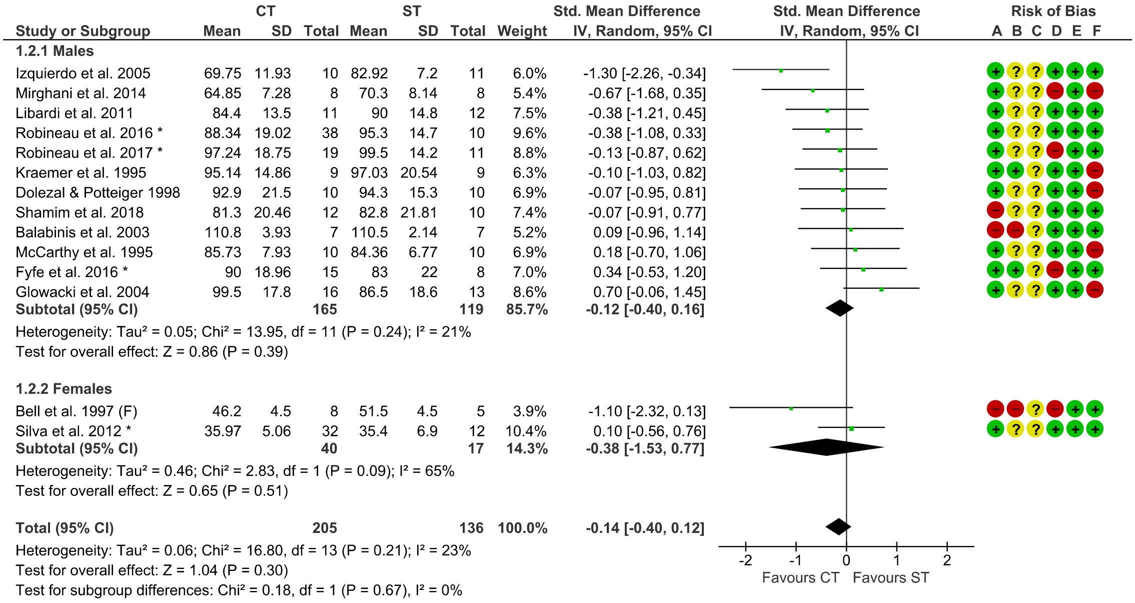

Risk of bias legend
(**A**) Random sequence generation (selection bias)
(**B**) Allocation concealment (selection bias)
(**C**) Blinding of outcome assessment (detection bias)
(**D**) Incomplete outcome data (attrition bias)
(**E**) Selective reporting (reporting bias)
(**F**) Other bias

Figure S2. Forest plot of studies comparing differences in adaptations in upper-body strength with concurrent training between males and females.

Study or Subgroup	CT Mean	CT SD	CT Total	ST Mean	ST SD	ST Total	Weight	Std. Mean Difference IV, Random, 95% CI
**1.3.1 Males**								
Prieto-González et al. 2022	35.16	1.29	10	37.07	1.81	10	5.8%	-1.16 [-2.13, -0.20]
Shamim et al. 2018	41.4	6.08	12	44.62	5.42	10	7.3%	-0.53 [-1.39, 0.32]
Chtara et al. 2008 *	285.62	14.9	20	290.6	11.29	9	8.6%	-0.35 [-1.14, 0.45]
Fyfe et al. 2016 *	48.87	7.97	15	52	12	8	7.2%	-0.32 [-1.18, 0.55]
Kraemer et al. 1995	697	112	9	735	123	9	6.2%	-0.31 [-1.24, 0.62]
Glowacki et al. 2004	52	11.6	16	54	8	13	10.0%	-0.19 [-0.93, 0.54]
Robineau et al. 2017 *	33.92	4.36	19	34.2	4.9	11	9.7%	-0.06 [-0.80, 0.68]
Robineau et al. 2016 *	34.93	4	27	35.2	6.9	10	10.2%	-0.05 [-0.78, 0.67]
Balabinis et al. 2003	59.6	2.37	7	58.7	1.88	7	4.8%	0.39 [-0.67, 1.46]
**Subtotal (95% CI)**			**135**			**87**	**69.8%**	**-0.27 [-0.55, 0.01]**
Heterogeneity: Tau² = 0.00; Chi² = 5.92, df = 8 (P = 0.66); I² = 0%								
Test for overall effect: Z = 1.92 (P = 0.05)								
**1.3.2 Females**								
Hendrickson et al. 2010	1,652.9	117.5	12	1,755.5	98.7	17	8.8%	-0.93 [-1.72, -0.15]
Spiliopoulou et al. 2021	24.91	4.58	9	29.06	4.42	10	5.9%	-0.88 [-1.84, 0.07]
Terzis et al. 2016	28.2	2.6	9	29.5	4.5	10	6.5%	-0.33 [-1.24, 0.58]
Laird et al. 2016	769.6	108.5	12	769.4	103.7	14	9.0%	0.00 [-0.77, 0.77]
**Subtotal (95% CI)**			**42**			**51**	**30.2%**	**-0.52 [-0.98, -0.06]**
Heterogeneity: Tau² = 0.03; Chi² = 3.55, df = 3 (P = 0.31); I² = 15%								
Test for overall effect: Z = 2.21 (P = 0.03)								
**Total (95% CI)**			**177**			**138**	**100.0%**	**-0.35 [-0.58, -0.11]**
Heterogeneity: Tau² = 0.00; Chi² = 10.35, df = 12 (P = 0.59); I² = 0%								
Test for overall effect: Z = 2.92 (P = 0.004)								
Test for subgroup differences: Chi² = 0.80, df = 1 (P = 0.37), I² = 0%								

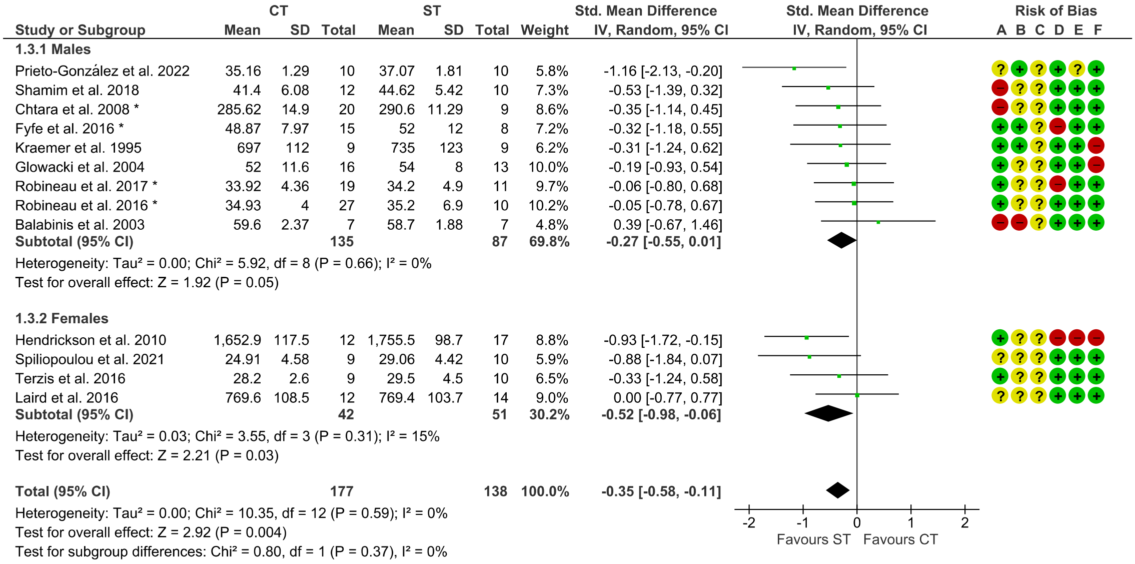

Risk of bias legend
(**A**) Random sequence generation (selection bias)
(**B**) Allocation concealment (selection bias)
(**C**) Blinding of outcome assessment (detection bias)
(**D**) Incomplete outcome data (attrition bias)
(**E**) Selective reporting (reporting bias)
(**F**) Other bias

Figure S3. Forest plot of studies comparing differences in adaptations in power with concurrent training between males and females.

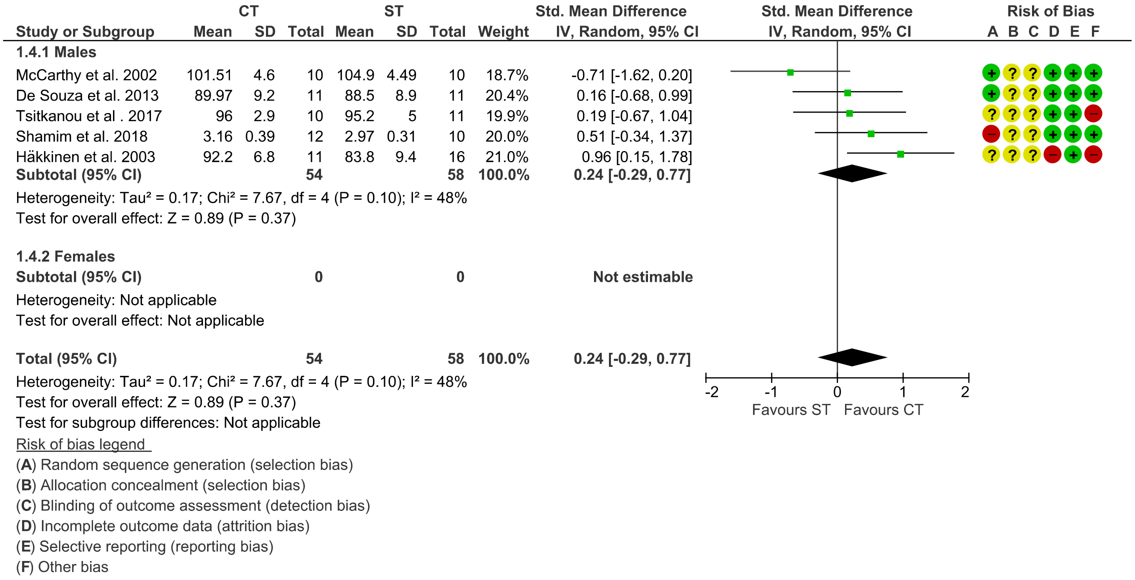

Figure S4. Forest plot of studies comparing differences in adaptations in muscle hypertrophy with concurrent training between males and females.

Study or Subgroup	CT Mean	CT SD	CT Total	ET Mean	ET SD	ET Total	Weight	Std. Mean Difference IV, Random, 95% CI	A	B	C	D	E	F
**1.5.1 Males**														
Nelson et al. 1990	48.8	2	5	54.7	5.5	4	1.2%	-1.34 [-2.90, 0.21]	+	?	?	−	+	−
Shaw et al. 2009	34.74	5.92	13	39.98	4.87	12	3.4%	-0.93 [-1.76, -0.10]	+	?	?	+	+	+
Libardi et al. 2012	34.43	5.4	11	38.55	3.98	12	3.2%	-0.84 [-1.70, 0.02]	+	?	?	+	+	+
McCarthy et al. 1995	45.5	5.69	10	48.7	8.22	10	3.1%	-0.43 [-1.32, 0.46]	+	?	?	+	+	−
Shamim et al. 2018	45.3	4.51	12	47.66	6.6	10	3.3%	-0.41 [-1.26, 0.44]	−	?	?	+	+	+
Damasceno et al. 2015	54.4	5.3	9	56.8	6	9	2.9%	-0.40 [-1.34, 0.53]	+	+	?	+	+	+
Kraemer et al. 1995	56.38	4.69	9	58.65	6.87	8	2.8%	-0.37 [-1.33, 0.59]	+	+	?	+	+	−
Dolezal & Potteiger 1998	55.8	5.2	10	57.1	5	10	3.1%	-0.24 [-1.12, 0.64]	+	?	?	+	+	−
Filipas et al. 2022 (1)	4.43	0.5	15	4.49	0.46	15	4.2%	-0.12 [-0.84, 0.59]	+	+	+	+	+	+
Levin et al. 2009	62.3	3.2	7	62.5	2.7	7	2.4%	-0.06 [-1.11, 0.98]	+	?	?	−	+	+
Vorup et al. 2016	59.8	5.6	8	60.1	5.5	6	2.4%	-0.05 [-1.11, 1.01]	+	?	?	+	+	+
Sánchez-Moreno et al. 2021 *	3.78	0.38	22	3.79	0.44	11	4.1%	-0.02 [-0.75, 0.70]	+	?	?	+	+	+
Millet et al. 2002	67.2	4.4	7	67.3	5.6	8	2.5%	-0.02 [-1.03, 1.00]	+	?	?	+	+	+
Beattie et al. 2017	59.1	2.78	6	59.07	2.27	9	2.5%	0.01 [-1.02, 1.04]	−	?	?	+	+	+
Bell et al. 2000 (M)	4.54	0.62	8	4.53	0.63	7	2.5%	0.02 [-1.00, 1.03]	+	?	?	+	+	+
Prieto-González et al. 2022	60.98	3.36	10	60.91	4.27	10	3.2%	0.02 [-0.86, 0.89]	?	+	?	+	?	+
Glowacki et al. 2004	44.6	6.8	16	44.3	7	12	3.9%	0.04 [-0.71, 0.79]	+	?	?	+	+	−
De Souza et al. 2013	53.8	8.62	11	52.4	5.31	8	3.0%	0.18 [-0.73, 1.09]	+	?	?	+	+	+
Gonçalves et al. 2022	48.8	8.11	10	46.4	12.43	11	3.3%	0.22 [-0.64, 1.08]	+	?	?	−	+	−
Skovgaard et al. 2014	59.5	2.77	12	58.2	6.9	9	3.2%	0.25 [-0.62, 1.12]	+	?	?	+	+	+
Hausswirth et al. 2010	70.8	5.5	7	68.3	10.1	7	2.4%	0.29 [-0.77, 1.34]	+	?	?	+	+	+
Aagaard et al . 2011	75	6	7	73	2.3	7	2.4%	0.41 [-0.65, 1.48]	+	?	?	+	+	+
Filipas et al. 2022 (2)	4.58	0.44	15	4.32	0.5	15	4.1%	0.54 [-0.19, 1.27]	+	+	+	+	+	+
Gómez-Molina et al. 2018	61.1	6.5	14	56.9	7.4	11	3.5%	0.59 [-0.22, 1.40]	+	?	?	+	+	+
Mikkola et al. 2012	43.3	7.3	11	39.4	3.9	11	3.2%	0.64 [-0.22, 1.50]	?	?	?	+	+	+
Chtara et al. 2005 *	57.44	2.09	20	54.73	3.42	10	3.5%	1.02 [0.21, 1.82]	−	?	?	+	+	+
Balabinis et al. 2003	62	1.91	7	59	0.9	7	1.6%	1.88 [0.55, 3.21]	−	−	?	+	+	+
**Subtotal (95% CI)**			**292**			**256**	**81.0%**	**0.04 [-0.17, 0.24]**						
Heterogeneity: Tau² = 0.08; Chi² = 36.28, df = 26 (P = 0.09); I² = 28%														
Test for overall effect: Z = 0.34 (P = 0.74)														
**1.5.2 Females**														
Johnston et al. 1997	48	2	6	51	1.9	6	1.6%	-1.42 [-2.75, -0.09]	+	?	?	+	+	+
Lemura et al. 2000	39.1	4.1	12	42.2	4.5	10	3.2%	-0.70 [-1.57, 0.17]	?	?	?	+	+	+
Štohanzl et al. 2018 *	36.68	6	20	38.3	4.5	11	4.0%	-0.29 [-1.03, 0.45]	+	?	?	−	+	+
Vikmoen et al 2016	52.5	4.2	11	53.5	1.8	8	3.0%	-0.28 [-1.20, 0.64]	+	?	?	−	+	+
Bell et al. 2000 (F)	3	0.2	5	3.05	0.6	4	1.7%	-0.11 [-1.42, 1.21]	+	?	?	+	+	+
Bishop et al. 1999	48.4	5.5	14	48.4	9.7	7	3.0%	0.00 [-0.91, 0.91]	+	?	?	+	+	+
Kelly et al. 2008	45.1	7.2	7	42.3	4.9	9	2.6%	0.44 [-0.56, 1.44]	+	?	?	+	+	+
**Subtotal (95% CI)**			**75**			**55**	**19.0%**	**-0.29 [-0.65, 0.08]**						
Heterogeneity: Tau² = 0.01; Chi² = 6.13, df = 6 (P = 0.41); I² = 2%														
Test for overall effect: Z = 1.54 (P = 0.12)														
**Total (95% CI)**			**367**			**311**	**100.0%**	**-0.03 [-0.21, 0.16]**						
Heterogeneity: Tau² = 0.08; Chi² = 44.93, df = 33 (P = 0.08); I² = 27%														
Test for overall effect: Z = 0.29 (P = 0.77)														
Test for subgroup differences: Chi² = 2.27, df = 1 (P = 0.13), I² = 55.9%														

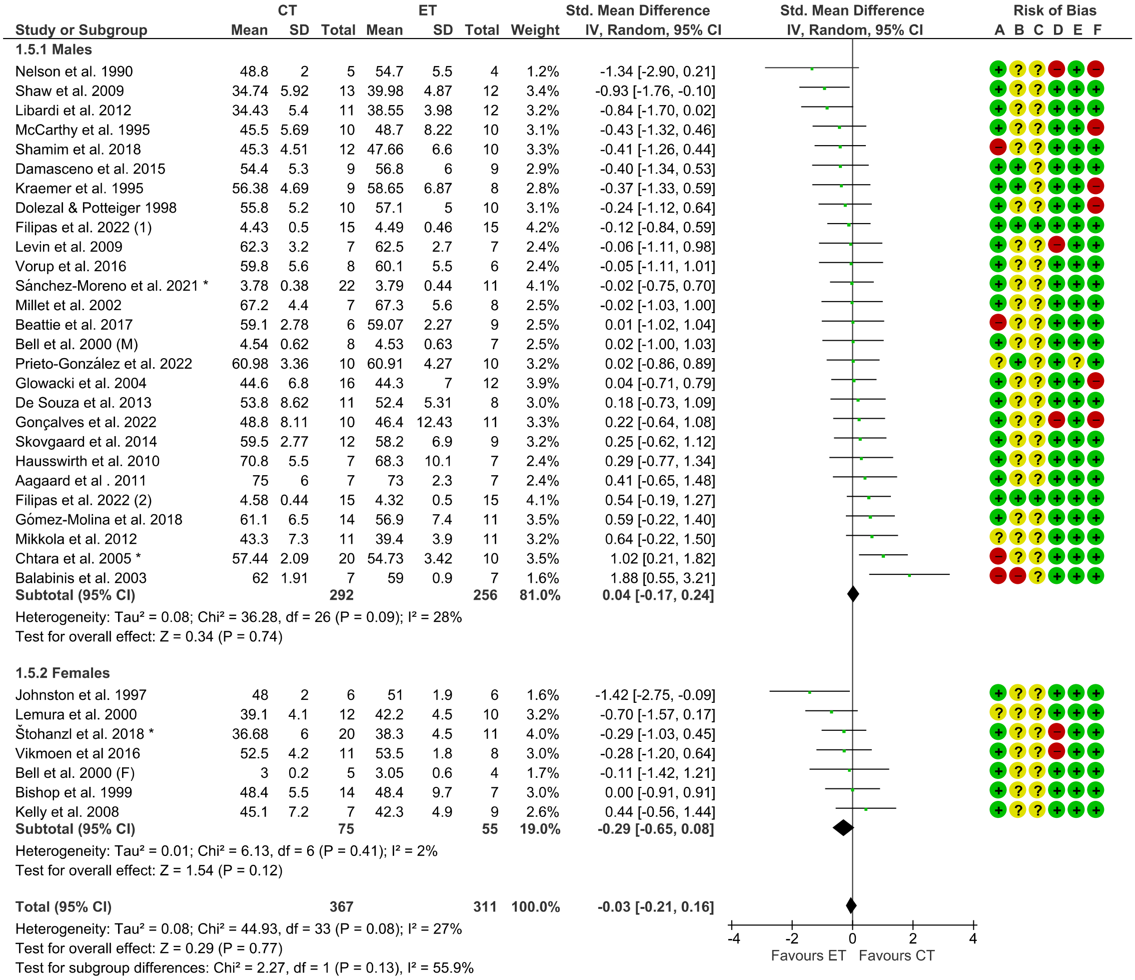

Risk of bias legend
(**A**) Random sequence generation (selection bias)
(**B**) Allocation concealment (selection bias)
(**C**) Blinding of outcome assessment (detection bias)
(**D**) Incomplete outcome data (attrition bias)
(**E**) Selective reporting (reporting bias)
(**F**) Other bias

Figure S5. Forest plot of studies comparing differences in adaptations in $\dot{V}O_{2max}$ with concurrent training between males and females.

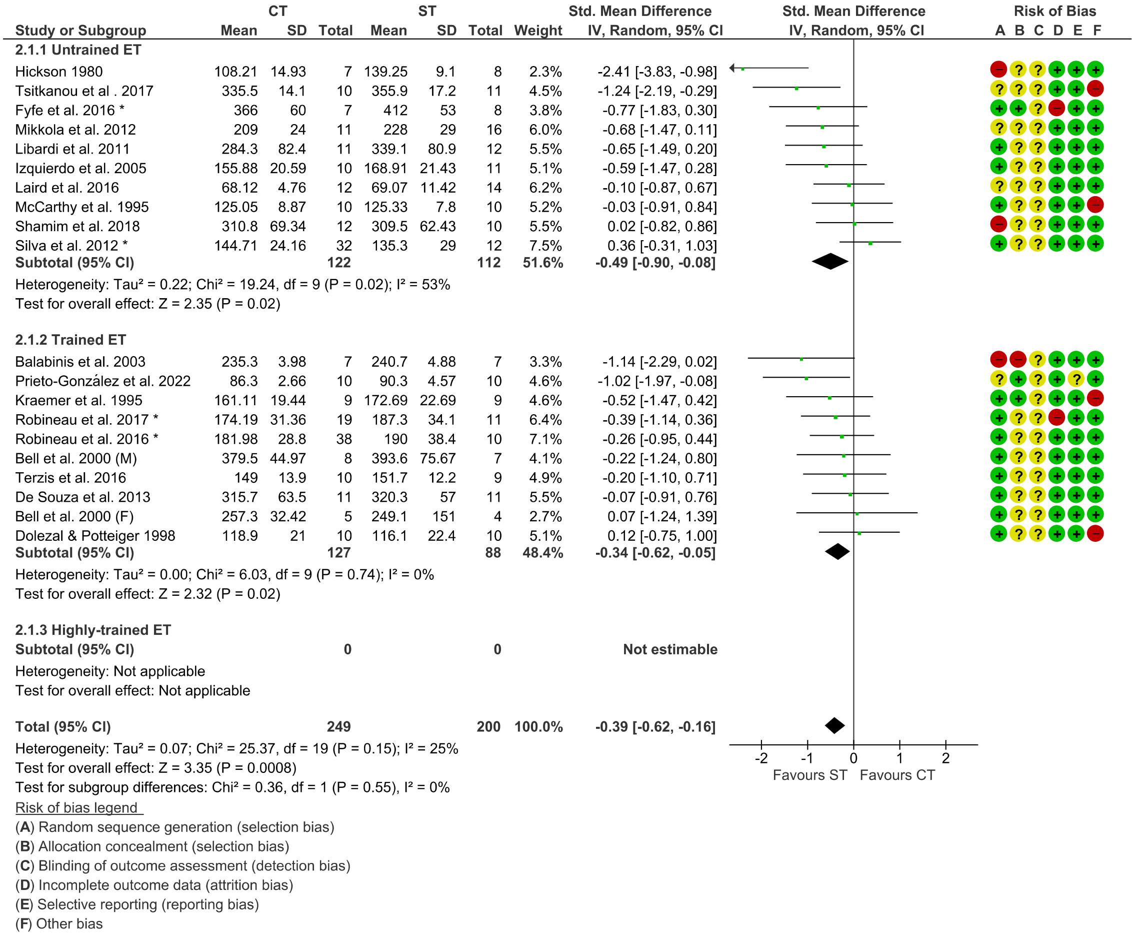

Figure S6. Forest plot of studies comparing differences in adaptations in lower-body strength with concurrent training between untrained and endurance-trained participants.

Study or Subgroup	CT Mean	CT SD	CT Total	ST Mean	ST SD	ST Total	Weight	Std. Mean Difference IV, Random, 95% CI	Risk of Bias A	B	C	D	E	F
**2.2.1 Untrained ET**														
Izquierdo et al. 2005	69.75	11.93	10	82.92	7.2	11	6.4%	-1.30 [-2.26, -0.34]	+	?	?	+	+	+
Libardi et al. 2011	84.4	13.5	11	90	14.8	12	8.1%	-0.38 [-1.21, 0.45]	+	?	?	+	+	+
Shamim et al. 2018	81.3	20.46	12	82.8	21.81	10	7.9%	-0.07 [-0.91, 0.77]	−	?	?	+	+	+
Silva et al. 2012 *	35.97	5.06	32	35.4	6.9	12	11.2%	0.10 [-0.56, 0.76]	+	?	?	+	+	+
McCarthy et al. 1995	85.73	7.93	10	84.36	6.77	10	7.4%	0.18 [-0.70, 1.06]	+	?	?	+	+	−
Fyfe et al. 2016 *	90	24	7	83	22	8	5.8%	0.29 [-0.73, 1.31]	+	+	?	−	+	+
Glowacki et al. 2004	99.5	17.8	16	86.5	18.6	13	9.3%	0.70 [-0.06, 1.45]	+	?	?	+	+	−
**Subtotal (95% CI)**			**98**			**76**	**56.1%**	**-0.04 [-0.48, 0.40]**						
Heterogeneity: Tau² = 0.17; Chi² = 11.65, df = 6 (P = 0.07); I² = 48%														
Test for overall effect: Z = 0.18 (P = 0.86)														
**2.2.2 Trained ET**														
Bell et al. 1997 (F)	46.2	4.5	8	51.5	4.5	5	4.2%	-1.10 [-2.32, 0.13]	−	−	?	−	+	+
Robineau et al. 2016 *	88.34	19.02	38	95.3	14.7	10	10.4%	-0.38 [-1.08, 0.33]	+	?	?	+	+	+
Robineau et al. 2017 *	97.24	18.75	19	99.5	14.2	11	9.6%	-0.13 [-0.87, 0.62]	+	?	?	−	+	+
Kraemer et al. 1995	95.14	14.86	9	97.03	20.54	9	6.8%	-0.10 [-1.03, 0.82]	+	+	?	+	+	−
Dolezal & Potteiger 1998	92.9	21.5	10	94.3	15.3	10	7.4%	-0.07 [-0.95, 0.81]	+	?	?	+	+	−
Balabinis et al. 2003	110.8	3.93	7	110.5	2.14	7	5.5%	0.09 [-0.96, 1.14]	−	−	?	+	+	+
**Subtotal (95% CI)**			**91**			**52**	**43.9%**	**-0.23 [-0.59, 0.12]**						
Heterogeneity: Tau² = 0.00; Chi² = 2.71, df = 5 (P = 0.74); I² = 0%														
Test for overall effect: Z = 1.29 (P = 0.20)														
**2.2.3 Highly-trained ET**														
**Subtotal (95% CI)**			**0**			**0**		**Not estimable**						
Heterogeneity: Not applicable														
Test for overall effect: Not applicable														
**Total (95% CI)**			**189**			**128**	**100.0%**	**-0.12 [-0.39, 0.15]**						
Heterogeneity: Tau² = 0.05; Chi² = 15.22, df = 12 (P = 0.23); I² = 21%														
Test for overall effect: Z = 0.87 (P = 0.39)														
Test for subgroup differences: Chi² = 0.45, df = 1 (P = 0.50), I² = 0%														

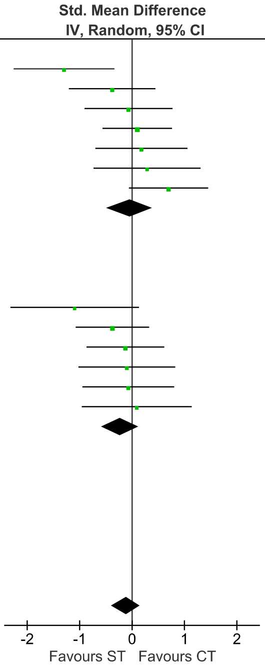

Risk of bias legend
(**A**) Random sequence generation (selection bias)
(**B**) Allocation concealment (selection bias)
(**C**) Blinding of outcome assessment (detection bias)
(**D**) Incomplete outcome data (attrition bias)
(**E**) Selective reporting (reporting bias)
(**F**) Other bias

Figure S7. Forest plot of studies comparing differences in adaptations in upper-body strength with concurrent training between untrained and endurance-trained participants.

Study or Subgroup	CT Mean	CT SD	CT Total	ST Mean	ST SD	ST Total	Weight	Std. Mean Difference IV, Random, 95% CI	Risk of Bias A	B	C	D	E	F
**2.3.1 Untrained ET**														
Spiliopoulou et al. 2021	24.91	4.58	9	29.06	4.42	10	6.0%	-0.88 [-1.84, 0.07]	?	?	?	+	+	+
Shamim et al. 2018	41.4	6.08	12	44.62	5.42	10	7.5%	-0.53 [-1.39, 0.32]	−	?	?	+	+	+
Glowacki et al. 2004	52	11.6	16	54	8	13	10.2%	-0.19 [-0.93, 0.54]	+	?	?	+	+	−
Fyfe et al. 2016 *	51	9	7	52	12	8	5.3%	-0.09 [-1.10, 0.93]	+	+	?	−	+	+
Laird et al. 2016	769.6	108.5	12	769.4	103.7	14	9.2%	0.00 [-0.77, 0.77]	?	?	?	+	+	+
**Subtotal (95% CI)**			**56**			**55**	**38.2%**	**-0.31 [-0.68, 0.07]**						
Heterogeneity: Tau² = 0.00; Chi² = 2.56, df = 4 (P = 0.63); I² = 0%														
Test for overall effect: Z = 1.58 (P = 0.11)														
**2.3.2 Trained ET**														
Prieto-González et al. 2022	35.16	1.29	10	37.07	1.81	10	5.9%	-1.16 [-2.13, -0.20]	?	+	?	+	?	+
Hendrickson et al. 2010	1,652.9	117.5	12	1,755.5	98.7	17	8.9%	-0.93 [-1.72, -0.15]	+	?	?	−	−	−
Chtara et al. 2008 *	285.62	14.9	20	290.6	11.29	9	8.7%	-0.35 [-1.14, 0.45]	−	?	?	+	+	+
Terzis et al. 2016	28.2	2.6	9	29.5	4.5	10	6.6%	-0.33 [-1.24, 0.58]	+	?	?	+	+	+
Kraemer et al. 1995	697	112	9	735	123	9	6.3%	-0.31 [-1.24, 0.62]	+	+	?	+	+	−
Robineau et al. 2017 *	33.92	4.36	19	34.2	4.9	11	9.9%	-0.06 [-0.80, 0.68]	+	?	?	−	+	+
Robineau et al. 2016 *	34.93	4	27	35.2	6.9	10	10.4%	-0.05 [-0.78, 0.67]	+	?	?	+	+	+
Balabinis et al. 2003	59.6	2.37	7	58.7	1.88	7	4.9%	0.39 [-0.67, 1.46]	−	−	?	+	+	+
**Subtotal (95% CI)**			**113**			**83**	**61.8%**	**-0.35 [-0.67, -0.03]**						
Heterogeneity: Tau² = 0.03; Chi² = 8.00, df = 7 (P = 0.33); I² = 12%														
Test for overall effect: Z = 2.16 (P = 0.03)														
**2.3.3 Highly-trained ET**														
**Subtotal (95% CI)**			**0**			**0**		**Not estimable**						
Heterogeneity: Not applicable														
Test for overall effect: Not applicable														
**Total (95% CI)**			**169**			**138**	**100.0%**	**-0.33 [-0.57, -0.10]**						
Heterogeneity: Tau² = 0.00; Chi² = 10.58, df = 12 (P = 0.56); I² = 0%														
Test for overall effect: Z = 2.79 (P = 0.005)														
Test for subgroup differences: Chi² = 0.03, df = 1 (P = 0.86), I² = 0%														

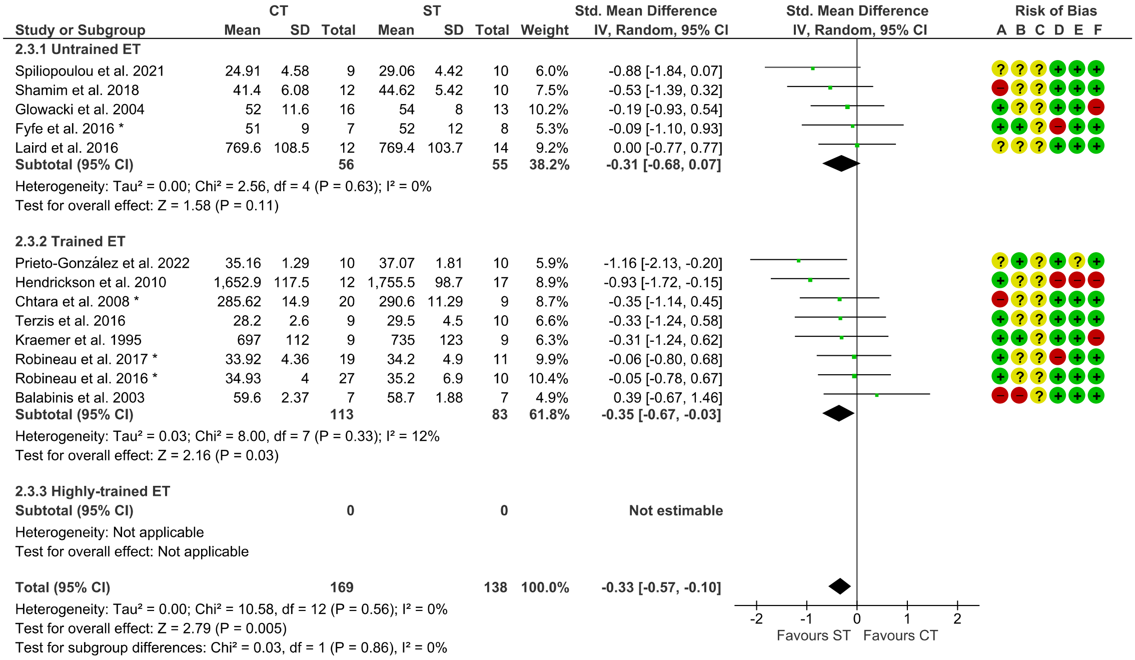

Risk of bias legend
(**A**) Random sequence generation (selection bias)
(**B**) Allocation concealment (selection bias)
(**C**) Blinding of outcome assessment (detection bias)
(**D**) Incomplete outcome data (attrition bias)
(**E**) Selective reporting (reporting bias)
(**F**) Other bias

Figure S8. Forest plot of studies comparing differences in adaptations in power with concurrent training between untrained and endurance-trained participants.

Study or Subgroup	CT Mean	CT SD	CT Total	ST Mean	ST SD	ST Total	Weight	Std. Mean Difference IV, Random, 95% CI
**2.4.1 Untrained ET**								
McCarthy et al. 2002	101.51	4.6	10	104.9	4.49	10	23.1%	-0.71 [-1.62, 0.20]
Tsitkanou et al . 2017	96	2.9	10	95.2	5	11	25.3%	0.19 [-0.67, 1.04]
Shamim et al. 2018	3.16	0.39	12	2.97	0.31	10	25.4%	0.51 [-0.34, 1.37]
**Subtotal (95% CI)**			**32**			**31**	**73.8%**	**0.01 [-0.70, 0.72]**
Heterogeneity: Tau² = 0.19; Chi² = 3.92, df = 2 (P = 0.14); I² = 49%								
Test for overall effect: Z = 0.03 (P = 0.98)								
**2.4.2 Trained ET**								
De Souza et al. 2013	89.97	9.2	11	88.5	8.9	11	26.2%	0.16 [-0.68, 0.99]
**Subtotal (95% CI)**			**11**			**11**	**26.2%**	**0.16 [-0.68, 0.99]**
Heterogeneity: Not applicable								
Test for overall effect: Z = 0.37 (P = 0.71)								
**2.4.3 Highly-trained ET**								
**Subtotal (95% CI)**			**0**			**0**		**Not estimable**
Heterogeneity: Not applicable								
Test for overall effect: Not applicable								
**Total (95% CI)**			**43**			**42**	**100.0%**	**0.05 [-0.45, 0.55]**
Heterogeneity: Tau² = 0.06; Chi² = 3.99, df = 3 (P = 0.26); I² = 25%								
Test for overall effect: Z = 0.21 (P = 0.83)								
Test for subgroup differences: Chi² = 0.07, df = 1 (P = 0.79), I² = 0%								

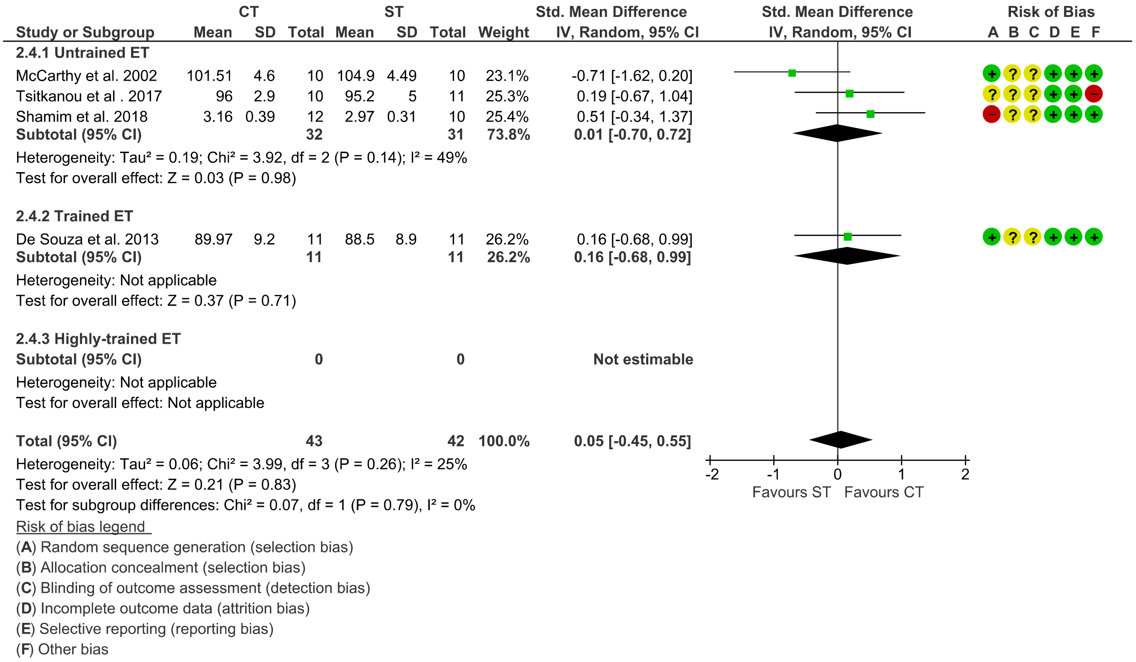

Risk of bias legend
(**A**) Random sequence generation (selection bias)
(**B**) Allocation concealment (selection bias)
(**C**) Blinding of outcome assessment (detection bias)
(**D**) Incomplete outcome data (attrition bias)
(**E**) Selective reporting (reporting bias)
(**F**) Other bias

Figure S9. Forest plot of studies comparing differences in adaptations in muscle hypertrophy with concurrent training between untrained and endurance-trained participants.

Study or Subgroup	CT Mean	CT SD	CT Total	ET Mean	ET SD	ET Total	Weight	Std. Mean Difference IV, Random, 95% CI
**2.5.1 untrained ET**								
Hickson 1980	47.1	1.7	7	50.9	3.4	8	1.7%	-1.30 [-2.45, -0.15]
Shaw et al. 2009	34.74	5.92	13	39.98	4.87	12	2.7%	-0.93 [-1.76, -0.10]
Libardi et al. 2012	34.43	5.4	11	38.55	3.98	12	2.6%	-0.84 [-1.70, 0.02]
Lemura et al. 2000	39.1	4.1	12	42.2	4.5	10	2.6%	-0.70 [-1.57, 0.17]
McCarthy et al. 1995	45.5	5.69	10	48.7	8.22	10	2.5%	-0.43 [-1.32, 0.46]
Shamim et al. 2018	45.3	4.51	12	47.66	6.6	10	2.6%	-0.41 [-1.26, 0.44]
Dudley et al. 1985	44.5	5.14	6	45.1	9.49	10	2.0%	-0.07 [-1.08, 0.94]
Glowacki et al. 2004	44.6	6.8	16	44.3	7	12	3.1%	0.04 [-0.71, 0.79]
Gonçalves et al. 2022	48.8	8.11	10	46.4	12.43	11	2.6%	0.22 [-0.64, 1.08]
Mikkola et al. 2012	43.3	7.3	11	39.4	3.9	11	2.6%	0.64 [-0.22, 1.50]
**Subtotal (95% CI)**			**108**			**106**	**25.0%**	**-0.35 [-0.70, 0.01]**
Heterogeneity: Tau² = 0.12; Chi² = 14.51, df = 9 (P = 0.11); I² = 38%								
Test for overall effect: Z = 1.92 (P = 0.05)								
**2.5.2 Trained ET**								
Damasceno et al. 2015	54.4	5.3	9	56.8	6	9	2.3%	-0.40 [-1.34, 0.53]
Kraemer et al. 1995	56.38	4.69	9	58.65	6.87	8	2.2%	-0.37 [-1.33, 0.59]
Dolezal & Potteiger 1998	55.8	5.2	10	57.1	5	10	2.5%	-0.24 [-1.12, 0.64]
Trowell et al. 2022	53.3	4.5	14	54.4	7.4	14	3.2%	-0.17 [-0.92, 0.57]
Bell et al. 2000 (F)	3	0.2	5	3.05	0.6	4	1.3%	-0.11 [-1.42, 1.21]
Levin et al. 2009	62.3	3.2	7	62.5	2.7	7	1.9%	-0.06 [-1.11, 0.98]
Vorup et al. 2016	59.8	5.6	8	60.1	5.5	6	1.9%	-0.05 [-1.11, 1.01]
Sánchez-Moreno et al. 2021 *	3.78	0.38	22	3.79	0.44	11	3.3%	-0.02 [-0.75, 0.70]
Bishop et al. 1999	48.4	5.5	14	48.4	9.7	7	2.4%	0.00 [-0.91, 0.91]
Beattie et al. 2017	59.1	2.78	6	59.07	2.27	9	2.0%	0.01 [-1.02, 1.04]
Bell et al. 2000 (M)	4.54	0.62	8	4.53	0.63	7	2.0%	0.02 [-1.00, 1.03]
Prieto-González et al. 2022	60.98	3.36	10	60.91	4.27	10	2.5%	0.02 [-0.86, 0.89]
De Souza et al. 2013	53.8	8.62	11	52.4	5.31	8	2.4%	0.18 [-0.73, 1.09]
Skovgaard et al. 2014	59.5	2.77	12	58.2	6.9	9	2.6%	0.25 [-0.62, 1.12]
Štohanzl et al. 2018 *	39.7	3	9	38.3	4.5	11	2.5%	0.34 [-0.55, 1.23]
Kelly et al. 2008	45.1	7.2	7	42.3	4.9	9	2.1%	0.44 [-0.56, 1.44]
Ferrauti et al. 2010	54.9	4.4	11	51.6	7.8	9	2.4%	0.51 [-0.38, 1.41]
Gómez-Molina et al. 2018	61.1	6.5	14	56.9	7.4	11	2.8%	0.59 [-0.22, 1.40]
Sunde et al. 2010	63.9	5.6	8	58	10.8	5	1.6%	0.69 [-0.47, 1.86]
Chtara et al. 2005 *	57.44	2.09	20	54.73	3.42	10	2.8%	1.02 [0.21, 1.82]
Balabinis et al. 2003	62	1.91	7	59	0.9	7	1.3%	1.88 [0.55, 3.21]
**Subtotal (95% CI)**			**221**			**181**	**48.2%**	**0.18 [-0.02, 0.38]**
Heterogeneity: Tau² = 0.00; Chi² = 18.95, df = 20 (P = 0.52); I² = 0%								
Test for overall effect: Z = 1.76 (P = 0.08)								
**2.5.3 Highly-trained ET**								
Johnston et al. 1997	48	2	6	51	1.9	6	1.3%	-1.42 [-2.75, -0.09]
Rønnestad et al. 2010b	69	2.4	6	72.5	2.7	6	1.4%	-1.26 [-2.55, 0.02]
Rønnestad et al. 2010a	69	1.6	11	69.8	2.5	9	2.5%	-0.37 [-1.26, 0.52]
Vikmoen et al 2016	52.5	4.2	11	53.5	1.8	8	2.4%	-0.28 [-1.20, 0.64]
Filipas et al. 2022 (1)	4.43	0.5	15	4.49	0.46	15	3.3%	-0.12 [-0.84, 0.59]
Millet et al. 2002	67.2	4.4	7	67.3	5.6	8	2.0%	-0.02 [-1.03, 1.00]
Losnegard et al 2011	64.86	2.45	9	64.8	2.36	10	2.4%	0.02 [-0.88, 0.92]
Hausswirth et al. 2010	70.8	5.5	7	68.3	10.1	7	1.9%	0.29 [-0.77, 1.34]
Aagaard et al . 2011	75	6	7	73	2.3	7	1.9%	0.41 [-0.65, 1.48]
Filipas et al. 2022 (2)	4.58	0.44	15	4.32	0.5	15	3.2%	0.54 [-0.19, 1.27]
Rønnestad et al. 2017	75	8	12	70	7	8	2.4%	0.63 [-0.29, 1.55]
Rønnestad et al. 2015	80	6	9	75	7	7	2.0%	0.73 [-0.30, 1.76]
**Subtotal (95% CI)**			**115**			**106**	**26.8%**	**0.01 [-0.32, 0.34]**
Heterogeneity: Tau² = 0.10; Chi² = 15.86, df = 11 (P = 0.15); I² = 31%								
Test for overall effect: Z = 0.07 (P = 0.94)								
**Total (95% CI)**			**444**			**393**	**100.0%**	**0.01 [-0.16, 0.17]**
Heterogeneity: Tau² = 0.08; Chi² = 58.00, df = 42 (P = 0.05); I² = 28%								
Test for overall effect: Z = 0.09 (P = 0.93)								
Test for subgroup differences: Chi² = 6.50, df = 2 (P = 0.04), I² = 69.2%								

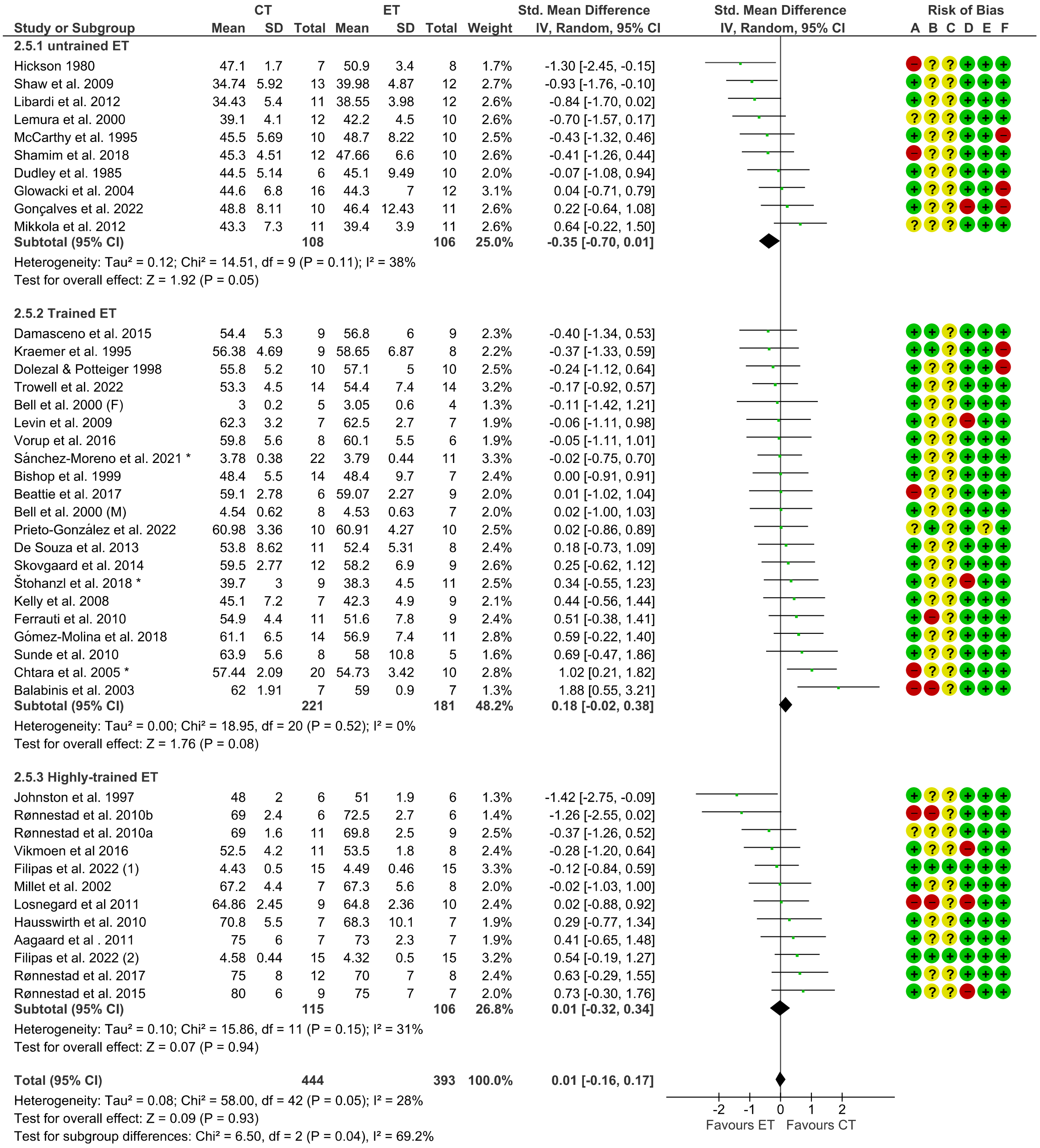

Risk of bias legend
(**A**) Random sequence generation (selection bias)
(**B**) Allocation concealment (selection bias)
(**C**) Blinding of outcome assessment (detection bias)
(**D**) Incomplete outcome data (attrition bias)
(**E**) Selective reporting (reporting bias)
(**F**) Other bias

Figure S10. Forest plot of studies comparing differences in adaptations in $\dot{V}O_{2max}$ with concurrent training between untrained, trained and highly-trained endurance athletes.

Study or Subgroup	CT Mean	CT SD	CT Total	ST Mean	ST SD	ST Total	Weight	Std. Mean Difference IV, Random, 95% CI	A	B	C	D	E	F
**2.6.1 Untrained ST**														
Prieto-González et al. 2022	86.3	2.66	10	90.3	4.57	10	5.1%	-1.02 [-1.97, -0.08]	?	+	?	+	?	+
Mikkola et al. 2012	209	24	11	228	29	16	7.2%	-0.68 [-1.47, 0.11]	?	?	?	+	+	+
Libardi et al. 2011	284.3	82.4	11	339.1	80.9	12	6.3%	-0.65 [-1.49, 0.20]	+	?	?	+	+	+
Fyfe et al. 2016 *	375.07	58.48	15	412	53	8	5.8%	-0.63 [-1.51, 0.25]	+	+	?	−	+	+
Izquierdo et al. 2005	155.88	20.59	10	168.91	21.43	11	5.8%	-0.59 [-1.47, 0.28]	+	?	?	+	+	+
Robineau et al. 2016 *	181.98	28.8	38	190	38.4	10	9.2%	-0.26 [-0.95, 0.44]	+	?	?	+	+	+
Laird et al. 2016	68.12	4.76	12	69.07	11.42	14	7.6%	-0.10 [-0.87, 0.67]	?	?	?	+	+	+
De Souza et al. 2013	315.7	63.5	11	320.3	57	11	6.4%	-0.07 [-0.91, 0.76]	+	?	?	+	+	+
McCarthy et al. 1995	125.05	8.87	10	125.33	7.8	10	5.9%	-0.03 [-0.91, 0.84]	+	?	?	+	+	−
Silva et al. 2012 *	144.71	24.16	32	135.3	29	12	10.1%	0.36 [-0.31, 1.03]	+	?	?	+	+	+
**Subtotal (95% CI)**			**160**			**114**	**69.4%**	**-0.31 [-0.57, -0.05]**						
Heterogeneity: Tau² = 0.01; Chi² = 9.43, df = 9 (P = 0.40); I² = 5%														
Test for overall effect: Z = 2.34 (P = 0.02)														
**2.6.2 Trained ST**														
Balabinis et al. 2003	235.3	3.98	7	240.7	4.88	7	3.4%	-1.14 [-2.29, 0.02]	−	−	?	+	+	+
Robineau et al. 2017 *	163.3	16.8	9	187.3	34.1	11	5.3%	-0.83 [-1.75, 0.10]	+	?	?	−	+	+
Kraemer et al. 1995	161.11	19.44	9	172.69	22.69	9	5.1%	-0.52 [-1.47, 0.42]	+	+	?	+	+	−
Shamim et al. 2018	310.8	69.34	12	309.5	62.43	10	6.4%	0.02 [-0.82, 0.86]	−	?	?	+	+	+
Dolezal & Potteiger 1998	118.9	21	10	116.1	22.4	10	5.9%	0.12 [-0.75, 1.00]	+	?	?	+	+	−
Mirghani et al. 2014	98.03	15.46	8	95.77	15.18	8	4.7%	0.14 [-0.84, 1.12]	+	?	?	−	+	−
**Subtotal (95% CI)**			**55**			**55**	**30.6%**	**-0.31 [-0.72, 0.10]**						
Heterogeneity: Tau² = 0.03; Chi² = 5.67, df = 5 (P = 0.34); I² = 12%														
Test for overall effect: Z = 1.48 (P = 0.14)														
**2.6.3 Highly-trained ST**														
**Subtotal (95% CI)**			**0**			**0**		**Not estimable**						
Heterogeneity: Not applicable														
Test for overall effect: Not applicable														
**Total (95% CI)**			**215**			**169**	**100.0%**	**-0.31 [-0.52, -0.09]**						
Heterogeneity: Tau² = 0.00; Chi² = 15.10, df = 15 (P = 0.44); I² = 1%														
Test for overall effect: Z = 2.83 (P = 0.005)														
Test for subgroup differences: Chi² = 0.00, df = 1 (P = 1.00), I² = 0%														

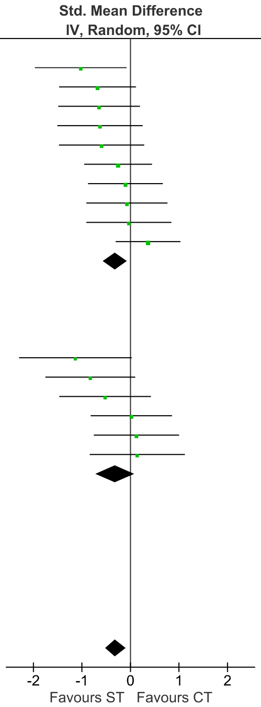

Risk of bias legend

(**A**) Random sequence generation (selection bias)
(**B**) Allocation concealment (selection bias)
(**C**) Blinding of outcome assessment (detection bias)
(**D**) Incomplete outcome data (attrition bias)
(**E**) Selective reporting (reporting bias)
(**F**) Other bias

Figure S11. Forest plot of studies comparing differences in adaptations in lower-body strength with concurrent training between untrained and strength-trained participants.

Study or Subgroup	CT Mean	CT SD	CT Total	ST Mean	ST SD	ST Total	Weight	Std. Mean Difference IV, Random, 95% CI	Risk of Bias A B C D E F
**2.7.1 Untrained ST**									
Izquierdo et al. 2005	69.75	11.93	10	82.92	7.2	11	6.2%	-1.30 [-2.26, -0.34]	+ ? ? + + +
Libardi et al. 2011	84.4	13.5	11	90	14.8	12	7.7%	-0.38 [-1.21, 0.45]	+ ? ? + + +
Robineau et al. 2016 *	88.34	19.02	38	95.3	14.7	10	9.7%	-0.38 [-1.08, 0.33]	+ ? ? + + +
Silva et al. 2012 *	35.97	5.06	32	35.4	6.9	12	10.4%	0.10 [-0.56, 0.76]	+ ? ? + + +
McCarthy et al. 1995	85.73	7.93	10	84.36	6.77	10	7.1%	0.18 [-0.70, 1.06]	+ ? ? + + −
Fyfe et al. 2016 *	90	18.96	15	83	22	8	7.2%	0.34 [-0.53, 1.20]	+ + ? − + +
Glowacki et al. 2004	99.5	17.8	16	86.5	18.6	13	8.7%	0.70 [-0.06, 1.45]	+ ? ? + + −
**Subtotal (95% CI)**			**132**			**76**	**56.9%**	**-0.08 [-0.52, 0.36]**	
Heterogeneity: Tau² = 0.19; Chi² = 12.88, df = 6 (P = 0.04); I² = 53%									
Test for overall effect: Z = 0.34 (P = 0.73)									
**2.7.2 Trained ST**									
Bell et al. 1997 (F)	46.2	4.5	8	51.5	4.5	5	4.1%	-1.10 [-2.32, 0.13]	− − ? − + +
Mirghani et al. 2014	64.85	7.28	8	70.3	8.14	8	5.6%	-0.67 [-1.68, 0.35]	+ ? ? − + −
Robineau et al. 2017 *	91.4	13.9	9	99.5	14.2	11	6.8%	-0.55 [-1.45, 0.35]	+ ? ? − + +
Kraemer et al. 1995	95.14	14.86	9	97.03	20.54	9	6.5%	-0.10 [-1.03, 0.82]	+ + ? + + −
Dolezal & Potteiger 1998	92.9	21.5	10	94.3	15.3	10	7.1%	-0.07 [-0.95, 0.81]	+ ? ? + + −
Shamim et al. 2018	81.3	20.46	12	82.8	21.81	10	7.5%	-0.07 [-0.91, 0.77]	− ? ? + + +
Balabinis et al. 2003	110.8	3.93	7	110.5	2.14	7	5.4%	0.09 [-0.96, 1.14]	− − ? + + +
**Subtotal (95% CI)**			**63**			**60**	**43.1%**	**-0.30 [-0.66, 0.06]**	
Heterogeneity: Tau² = 0.00; Chi² = 3.68, df = 6 (P = 0.72); I² = 0%									
Test for overall effect: Z = 1.62 (P = 0.11)									
**2.7.3 Highly-trained ST**									
**Subtotal (95% CI)**			**0**			**0**		**Not estimable**	
Heterogeneity: Not applicable									
Test for overall effect: Not applicable									
**Total (95% CI)**			**195**			**136**	**100.0%**	**-0.17 [-0.44, 0.10]**	
Heterogeneity: Tau² = 0.07; Chi² = 17.60, df = 13 (P = 0.17); I² = 26%									
Test for overall effect: Z = 1.23 (P = 0.22)									
Test for subgroup differences: Chi² = 0.57, df = 1 (P = 0.45), I² = 0%									

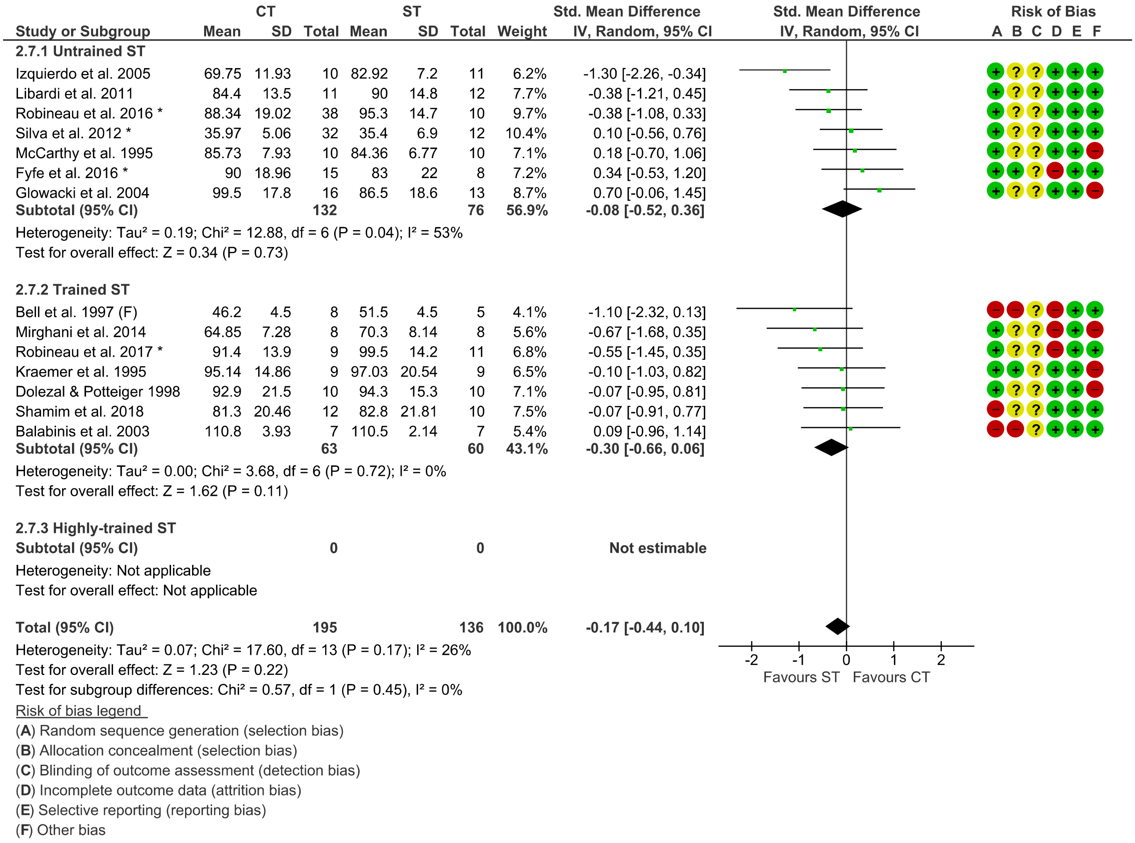

Risk of bias legend

(**A**) Random sequence generation (selection bias)
(**B**) Allocation concealment (selection bias)
(**C**) Blinding of outcome assessment (detection bias)
(**D**) Incomplete outcome data (attrition bias)
(**E**) Selective reporting (reporting bias)
(**F**) Other bias

Figure S12. Forest plot of studies comparing differences in adaptations in upper-body strength with concurrent training between untrained and strength-trained participants.

Study or Subgroup	CT Mean	CT SD	CT Total	RT Mean	RT SD	RT Total	Weight	Std. Mean Difference IV, Random, 95% CI	A	B	C	D	E	F
**2.8.1 Untrained ST**														
Prieto-González et al. 2022	35.16	1.29	10	37.07	1.81	10	7.0%	-1.16 [-2.13, -0.20]	?	+	?	+	?	+
Hendrickson et al. 2010	1,652.9	117.5	12	1,755.5	98.7	17	10.7%	-0.93 [-1.72, -0.15]	+	?	?	−	−	−
Spiliopoulou et al. 2021	24.91	4.58	9	29.06	4.42	10	7.2%	-0.88 [-1.84, 0.07]	?	?	?	+	+	+
Fyfe et al. 2016 *	48.87	7.97	15	52	12	8	8.8%	-0.32 [-1.18, 0.55]	+	+	?	−	+	+
Glowacki et al. 2004	52	11.6	16	54	8	13	12.2%	-0.19 [-0.93, 0.54]	+	?	?	+	+	−
Robineau et al. 2016 *	34.93	4	27	35.2	6.9	10	12.4%	-0.05 [-0.78, 0.67]	+	?	?	+	+	+
Laird et al. 2016	769.6	108.5	12	769.4	103.7	14	11.0%	0.00 [-0.77, 0.77]	?	?	?	+	+	+
**Subtotal (95% CI)**			**101**			**82**	**69.3%**	**-0.45 [-0.79, -0.11]**						
Heterogeneity: Tau² = 0.04; Chi² = 7.39, df = 6 (P = 0.29); I² = 19%														
Test for overall effect: Z = 2.57 (P = 0.01)														
**2.8.2 Trained ST**														
Shamim et al. 2018	41.4	6.08	12	44.62	5.42	10	8.9%	-0.53 [-1.39, 0.32]	−	?	?	+	+	+
Kraemer et al. 1995	697	112	9	735	123	9	7.6%	-0.31 [-1.24, 0.62]	+	+	?	+	+	−
Robineau et al. 2017 *	33.6	4.2	9	34.2	4.9	11	8.4%	-0.12 [-1.01, 0.76]	+	?	?	−	+	+
Balabinis et al. 2003	59.6	2.37	7	58.7	1.88	7	5.8%	0.39 [-0.67, 1.46]	−	−	?	+	+	+
**Subtotal (95% CI)**			**37**			**37**	**30.7%**	**-0.19 [-0.65, 0.27]**						
Heterogeneity: Tau² = 0.00; Chi² = 1.87, df = 3 (P = 0.60); I² = 0%														
Test for overall effect: Z = 0.81 (P = 0.42)														
**2.8.3 Highly-trained ST**														
**Subtotal (95% CI)**			**0**			**0**		**Not estimable**						
Heterogeneity: Not applicable														
Test for overall effect: Not applicable														
**Total (95% CI)**			**138**			**119**	**100.0%**	**-0.36 [-0.62, -0.11]**						
Heterogeneity: Tau² = 0.00; Chi² = 10.01, df = 10 (P = 0.44); I² = 0%														
Test for overall effect: Z = 2.77 (P = 0.006)														
Test for subgroup differences: Chi² = 0.77, df = 1 (P = 0.38), I² = 0%														

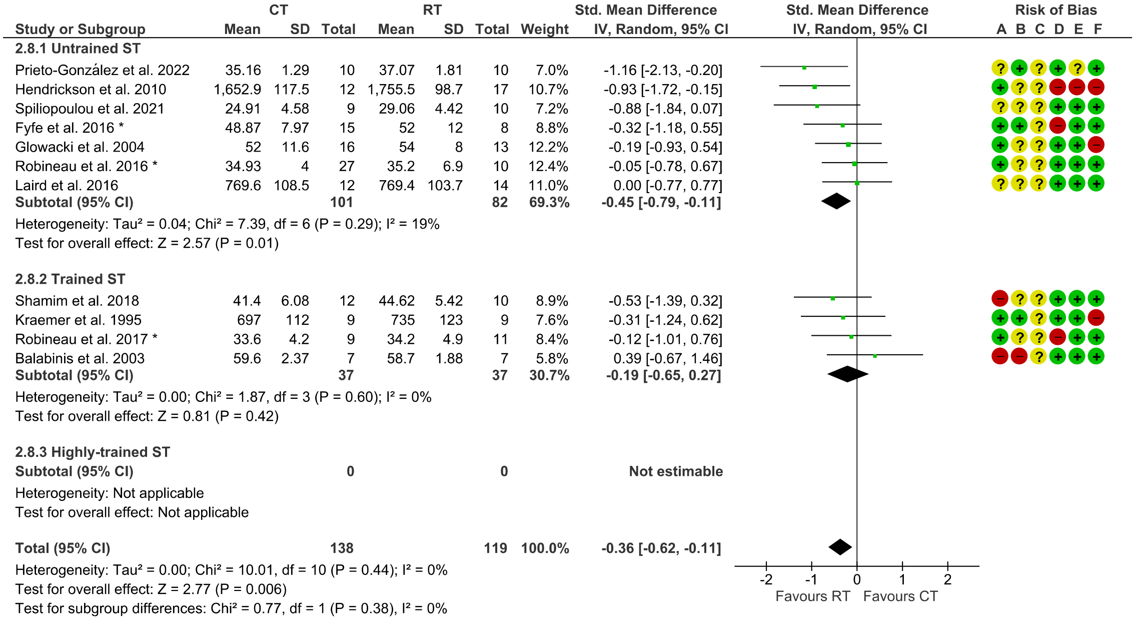

Risk of bias legend
(**A**) Random sequence generation (selection bias)
(**B**) Allocation concealment (selection bias)
(**C**) Blinding of outcome assessment (detection bias)
(**D**) Incomplete outcome data (attrition bias)
(**E**) Selective reporting (reporting bias)
(**F**) Other bias

Figure S13. Forest plot of studies comparing differences in adaptations in power with concurrent training between untrained and strength-trained participants.

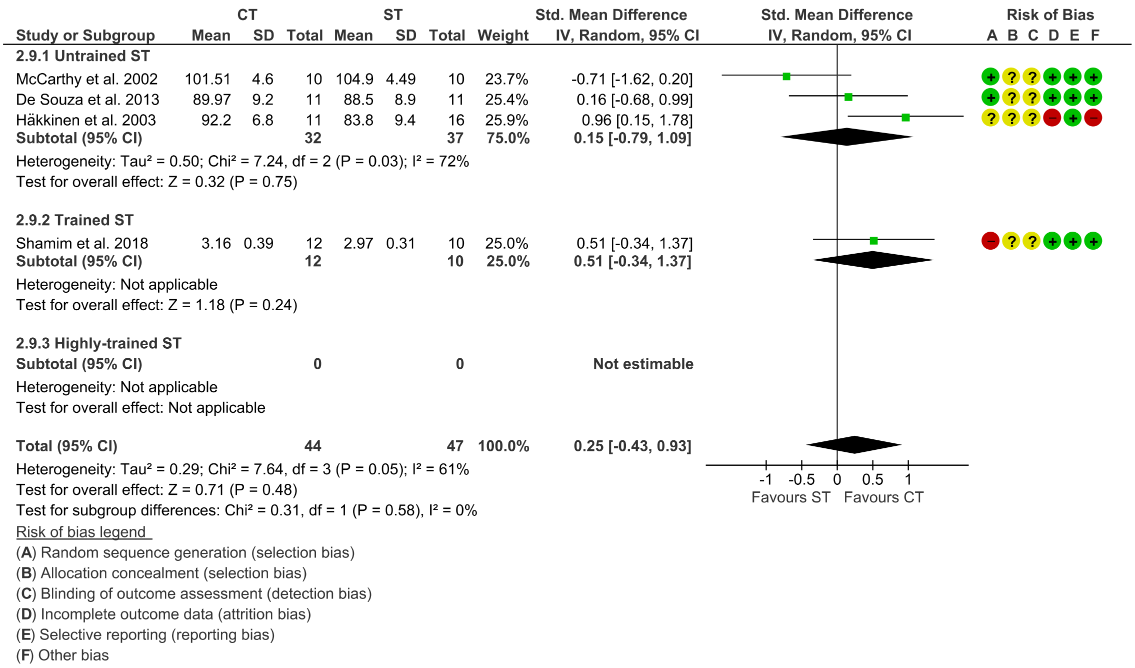

Figure S14. Forest plot of studies comparing differences in adaptations in muscle hypertrophy with concurrent training between untrained and strength-trained participants.

Study or Subgroup	CT Mean	CT SD	CT Total	ET Mean	ET SD	ET Total	Weight	Std. Mean Difference IV, Random, 95% CI	Risk of Bias A	B	C	D	E	F
**2.10.1 Untrained ST**														
Nelson et al. 1990	48.8	2	5	54.7	5.5	4	1.7%	-1.34 [-2.90, 0.21]	+	?	?	–	+	–
Hickson 1980	47.1	1.7	7	50.9	3.4	8	2.9%	-1.30 [-2.45, -0.15]	–	?	?	+	+	+
Libardi et al. 2012	34.43	5.4	11	38.55	3.98	12	4.4%	-0.84 [-1.70, 0.02]	+	?	?	+	+	+
McCarthy et al. 1995	45.5	5.69	10	48.7	8.22	10	4.2%	-0.43 [-1.32, 0.46]	+	?	?	+	+	–
Damasceno et al. 2015	54.4	5.3	9	56.8	6	9	3.9%	-0.40 [-1.34, 0.53]	+	+	?	+	+	+
Štohanzl et al. 2018 *	36.68	6	20	38.3	4.5	11	5.4%	-0.29 [-1.03, 0.45]	+	?	?	–	+	+
Vikmoen et al 2016	52.5	4.2	11	53.5	1.8	8	4.1%	-0.28 [-1.20, 0.64]	+	?	?	–	+	+
Trowell et al. 2022	53.3	4.5	14	54.4	7.4	14	5.4%	-0.17 [-0.92, 0.57]	+	?	?	+	+	+
Vorup et al. 2016	59.8	5.6	8	60.1	5.5	6	3.3%	-0.05 [-1.11, 1.01]	+	?	?	+	+	+
Beattie et al. 2017	59.1	2.78	6	59.07	2.27	9	3.4%	0.01 [-1.02, 1.04]	–	?	?	+	+	+
Prieto-González et al. 2022	60.98	3.36	10	60.91	4.27	10	4.3%	0.02 [-0.86, 0.89]	?	+	?	+	?	+
Glowacki et al. 2004	44.6	6.8	16	44.3	7	12	5.3%	0.04 [-0.71, 0.79]	+	?	?	+	+	–
De Souza et al. 2013	53.8	8.62	11	52.4	5.31	8	4.1%	0.18 [-0.73, 1.09]	+	?	?	+	+	+
Kelly et al. 2008	45.1	7.2	7	42.3	4.9	9	3.6%	0.44 [-0.56, 1.44]	+	?	?	+	+	+
Ferrauti et al. 2010	54.9	4.4	11	51.6	7.8	9	4.2%	0.51 [-0.38, 1.41]	+	–	?	+	+	+
Gômez-Molina et al. 2018	61.1	6.5	14	56.9	7.4	11	4.8%	0.59 [-0.22, 1.40]	+	?	?	+	+	+
Mikkola et al. 2012	43.3	7.3	11	39.4	3.9	11	4.4%	0.64 [-0.22, 1.50]	?	?	?	+	+	+
**Subtotal (95% CI)**			**181**			**161**	**69.6%**	**-0.09 [-0.34, 0.16]**						
Heterogeneity: Tau² = 0.05; Chi² = 20.01, df = 16 (P = 0.22); I² = 20%														
Test for overall effect: Z = 0.72 (P = 0.47)														
**2.10.2 Trained ST**														
Johnston et al. 1997	48	2	6	51	1.9	6	2.3%	-1.42 [-2.75, -0.09]	+	?	?	+	+	+
Shamim et al. 2018	45.3	4.51	12	47.66	6.6	10	4.5%	-0.41 [-1.26, 0.44]	–	?	?	+	+	+
Kraemer et al. 1995	56.38	4.69	9	58.65	6.87	8	3.8%	-0.37 [-1.33, 0.59]	+	+	?	+	+	–
Dolezal & Potteiger 1998	55.8	5.2	10	57.1	5	10	4.3%	-0.24 [-1.12, 0.64]	+	?	?	+	+	–
Levin et al. 2009	62.3	3.2	7	62.5	2.7	7	3.3%	-0.06 [-1.11, 0.98]	+	?	?	–	+	+
Sânchez-Moreno et al. 2021 *	3.78	0.38	22	3.79	0.44	11	5.5%	-0.02 [-0.75, 0.70]	+	?	?	+	+	+
Skovgaard et al. 2014	59.5	2.77	12	58.2	6.9	9	4.4%	0.25 [-0.62, 1.12]	+	?	?	+	+	+
Balabinis et al. 2003	62	1.91	7	59	0.9	7	2.3%	1.88 [0.55, 3.21]	–	–	?	+	+	+
**Subtotal (95% CI)**			**85**			**68**	**30.4%**	**-0.08 [-0.56, 0.40]**						
Heterogeneity: Tau² = 0.23; Chi² = 13.89, df = 7 (P = 0.05); I² = 50%														
Test for overall effect: Z = 0.31 (P = 0.76)														
**2.10.3 Highly-trained ST**														
**Subtotal (95% CI)**			**0**			**0**		**Not estimable**						
Heterogeneity: Not applicable														
Test for overall effect: Not applicable														
**Total (95% CI)**			**266**			**229**	**100.0%**	**-0.09 [-0.31, 0.13]**						
Heterogeneity: Tau² = 0.09; Chi² = 33.90, df = 24 (P = 0.09); I² = 29%														
Test for overall effect: Z = 0.81 (P = 0.42)														
Test for subgroup differences: Chi² = 0.00, df = 1 (P = 0.96), I² = 0%														

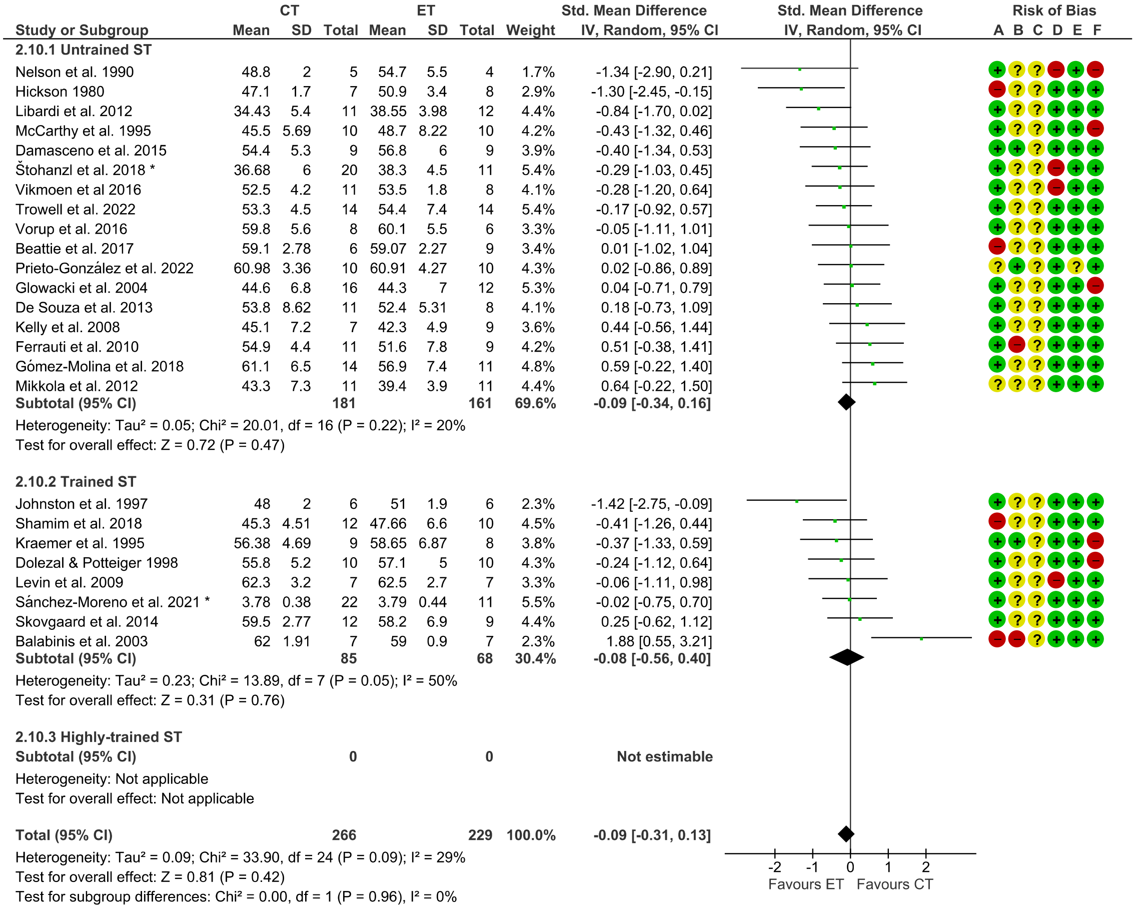

Risk of bias legend
(**A**) Random sequence generation (selection bias)
(**B**) Allocation concealment (selection bias)
(**C**) Blinding of outcome assessment (detection bias)
(**D**) Incomplete outcome data (attrition bias)
(**E**) Selective reporting (reporting bias)
(**F**) Other bias

Figure S15. Forest plot of studies comparing differences in adaptations in $\dot{V}O_{2max}$ with concurrent training between untrained and strength-trained participants.

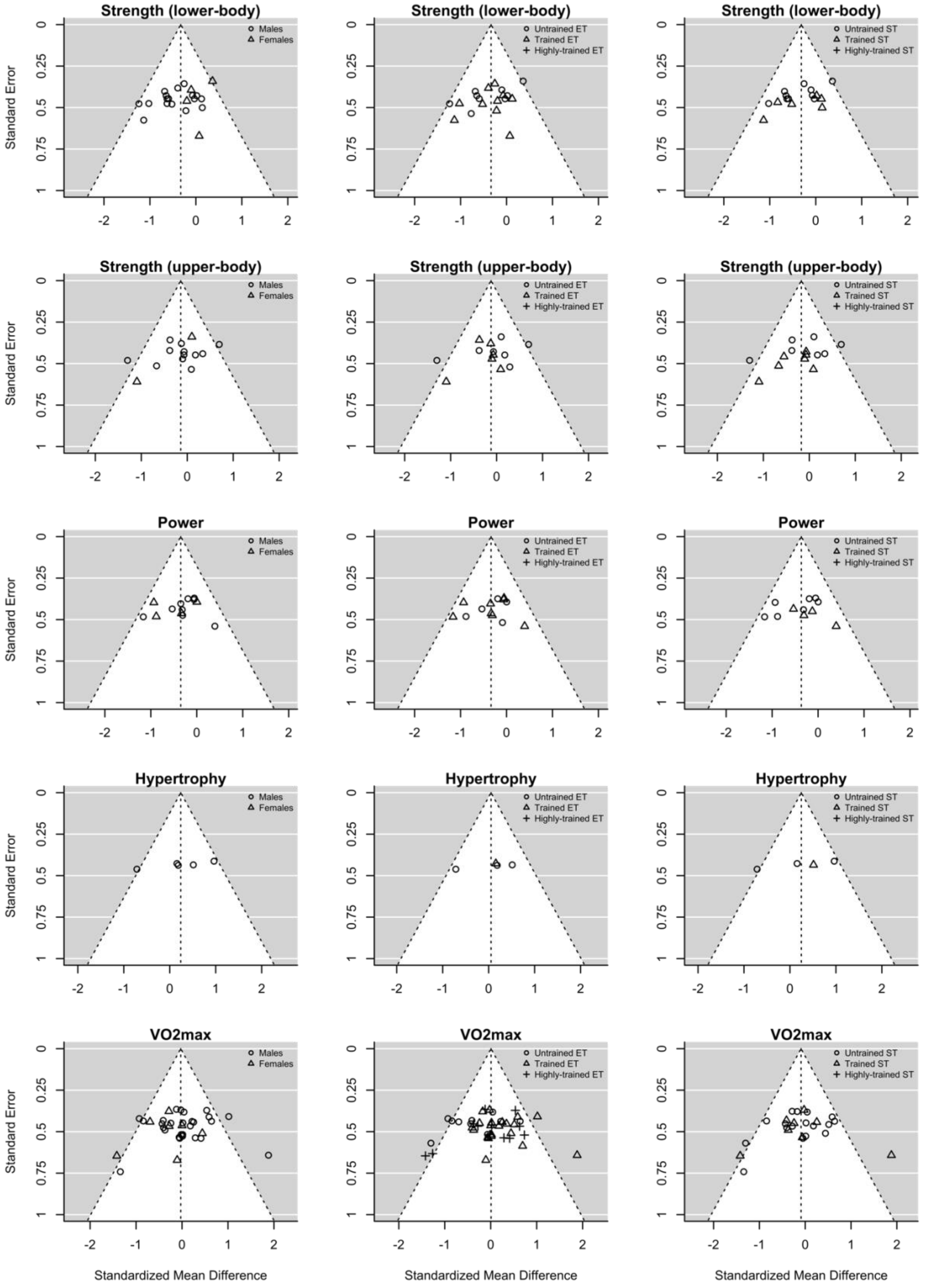

Figure S16. Funnel plots for outcome measures maximal lower-body and upper-body strength, power, muscle hypertrophy and $\dot{V}O_{2max}$ with comparisons related to sex (left panels), endurance training status (middle panels) and strength training status (right panels). Egger's regression tests showed no funnel plot asymmetries indicative for a publication bias (*P*>0.05), except for lower-body strength with levels of strength training status (*P*=0.033).

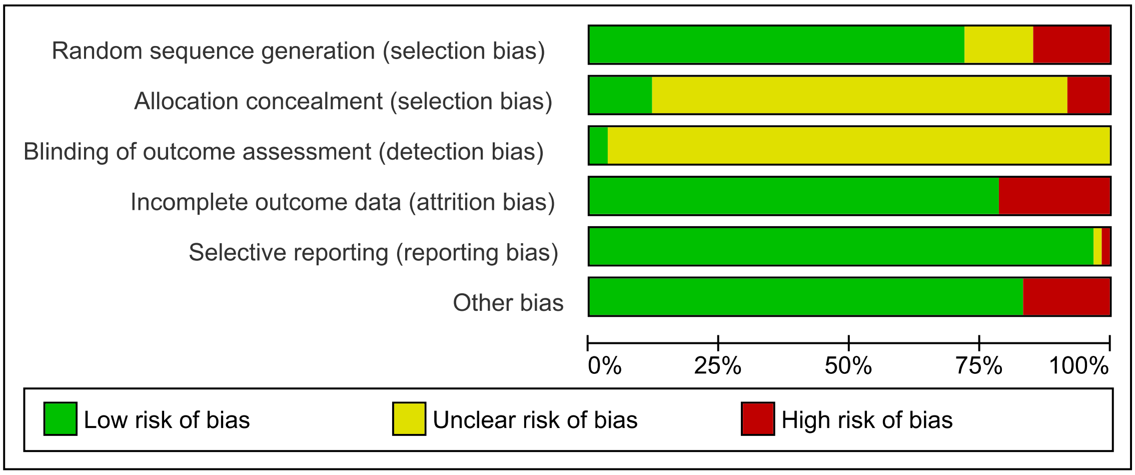

Figure S17. Risk of bias assessment of all included studies according to the Cochrane guidelines.

References

1. Chtara M, Chaouachi A, Levin GT, Chaouachi M, Chamari K, Amri M, et al. Effect of concurrent endurance and circuit resistance training sequence on muscular strength and power development. J Strength Cond Res. 2008;22:1037–45.

2. Chtara M, Chamari K, Chaouachi M, Chaouachi A, Koubaa D, Feki Y, et al. Effects of intra-session concurrent endurance and strength training sequence on aerobic performance and capacity. Br J Sports Med. 2005;39:555–60.

3. Libardi CA, De Souza GV, Cavaglieri CR, Madruga VA, Chacon-Mikahil MPT. Effect of resistance, endurance, and concurrent training on TNF-α, IL-6, and CRP. Med Sci Sports Exerc. 2012;44:50–6.

4. Libardi CA, Souza GV, Gáspari AF, Dos Santos CF, Leite ST, Dias R, et al. Effects of concurrent training on interleukin-6, tumour necrosis factor-alpha and C-reactive protein in middle-aged men. J Sports Sci. 2011;29:1573–81.

5. McCarthy JP, Pozniak MA, Agre JC. Neuromuscular adaptations to concurrent strength and endurance training. Med Sci Sports Exerc. 2002;34:511–9.

6. McCarthy JP, Agre JC, Graf BK, Pozniak MA, Vailas AC. Compatibility of adaptive responses with combining strength and endurance training. Med Sci Sports Exerc. 1995;27:429–36.

7. de Souza EO, Tricoli V, Aoki MS, Roschel H, Brum PC, Bacurau AVN, et al. Effects of concurrent strength and endurance training on genes related to myostatin signaling pathway and muscle fiber responses. J Strength Cond Res. 2014;28:3215–23.

8. de Souza EO, Tricoli V, Roschel H, Brum PC, Bacurau AVN, Ferreira JCB, et al. Molecular adaptations to concurrent training. Int J Sports Med. 2013;34:207–13.

9. Kraemer WJ, Ratamess NA. Fundamentals of resistance training: progression and exercise prescription. Med Sci Sports Exerc. 2004;36:674–88.

10. Kraemer WJ, Patton JF, Gordon SE, Harman EA, Deschenes MR, Reynolds K, et al. Compatibility of high-intensity strength and endurance training on hormonal and skeletal muscle adaptations. J Appl Physiol (1985). 1995;78:976–89.

11. Rønnestad BR, Hansen EA, Raastad T. Strength training improves 5-min all-out performance following 185 min of cycling. Scand J Med Sci Sports. 2011;21:250–9.

12. Rønnestad BR, Hansen EA, Raastad T. Effect of heavy strength training on thigh muscle cross-sectional area, performance determinants, and performance in well-trained cyclists. Eur J Appl Physiol. 2010a;108:965–75.

13. Rønnestad BR, Hansen J, Hollan I, Spencer M, Ellefsen S. Impairment of Performance Variables After In-Season Strength-Training Cessation in Elite Cyclists. Int J Sports Physiol Perform. 2016;11:727–35.

14. Rønnestad BR, Hansen J, Hollan I, Ellefsen S. Strength training improves performance and pedaling characteristics in elite cyclists. Scand J Med Sci Sports. 2015;25:e89-98.

15. Vikmoen O, Raastad T, Seynnes O, Bergstrøm K, Ellefsen S, Rønnestad BR. Effects of Heavy Strength Training on Running Performance and Determinants of Running Performance in Female Endurance Athletes. PLoS One. 2016;11:e0150799.

16. Vikmoen O, Ellefsen S, Trøen Ø, Hollan I, Hanestadhaugen M, Raastad T, et al. Strength training improves cycling performance, fractional utilization of VO2max and cycling economy in female cyclists. Scand J Med Sci Sports. 2016;26:384–96.

17. Beattie K, Carson BP, Lyons M, Rossiter A, Kenny IC. The Effect of Strength Training on Performance Indicators in Distance Runners. J Strength Cond Res. 2017;31:9–23.

18. Gergley JC. Comparison of two lower-body modes of endurance training on lower-body strength development while concurrently training. J Strength Cond Res. 2009;23:979–87.

19. Gravelle BL, Blessing DL. Physiological Adaptation in Women Concurrently Training for Strength and Endurance. The Journal of Strength & Conditioning Research. 2000;14:5.

20. Jones TW, Howatson G, Russell M, French DN. Performance and Endocrine Responses to Differing Ratios of Concurrent Strength and Endurance Training. J Strength Cond Res. 2016;30:693–702.

21. Kawano H, Fujimoto K, Higuchi M, Miyachi M. Effect of combined resistance and aerobic training on reactive hyperemia in men. J Physiol Sci. 2009;59:457–64.

22. Lee MJ-C, Ballantyne JK, Chagolla J, Hopkins WG, Fyfe JJ, Phillips SM, et al. Order of same-day concurrent training influences some indices of power development, but not strength, lean mass, or aerobic fitness in healthy, moderately-active men after 9 weeks of training. PLoS One. 2020;15:e0233134.

23. Leveritt M, Abernethy PJ, Barry B, Logan PA. Concurrent strength and endurance training: the influence of dependent variable selection. J Strength Cond Res. 2003;17:503–8.

24. Psilander N, Frank P, Flockhart M, Sahlin K. Adding strength to endurance training does not enhance aerobic capacity in cyclists. Scand J Med Sci Sports. 2015;25:e353-359.

25. Støren O, Helgerud J, Støa EM, Hoff J. Maximal strength training improves running economy in distance runners. Med Sci Sports Exerc. 2008;40:1087–92.

26. Timmins RG, Shamim B, Tofari PJ, Hickey JT, Camera DM. Differences in Lower Limb Strength and Structure After 12 Weeks of Resistance, Endurance, and Concurrent Training. Int J Sports Physiol Perform. 2020;1–8.

27. Aagaard P, Andersen JL, Bennekou M, Larsson B, Olesen JL, Crameri R, et al. Effects of resistance training on endurance capacity and muscle fiber composition in young top-level cyclists. Scand J Med Sci Sports. 2011;21:e298-307.

28. Balabinis CP, Psarakis CH, Moukas M, Vassiliou MP, Behrakis PK. Early phase changes by concurrent endurance and strength training. J Strength Cond Res. 2003;17:393–401.

29. Beattie K, Carson BP, Lyons M, Kenny IC. The Effect of Maximal- and Explosive-Strength Training on Performance Indicators in Cyclists. Int J Sports Physiol Perform. 2017;12:470–80.

30. Bell G, Syrotuik D, Socha T, Maclean I, Quinney HA. Effect of Strength Training and Concurrent Strength and Endurance Training on Strength, Testosterone, and Cortisol. The Journal of Strength & Conditioning Research. 1997;11:57.

31. Bell GJ, Syrotuik D, Martin TP, Burnham R, Quinney HA. Effect of concurrent strength and endurance training on skeletal muscle properties and hormone concentrations in humans. Eur J Appl Physiol. 2000;81:418–27.

32. Bishop D, Jenkins DG, Mackinnon LT, McEniery M, Carey MF. The effects of strength training on endurance performance and muscle characteristics. Med Sci Sports Exerc. 1999;31:886–91.

33. Damasceno MV, Lima-Silva AE, Pasqua LA, Tricoli V, Duarte M, Bishop DJ, et al. Effects of resistance training on neuromuscular characteristics and pacing during 10-km running time trial. Eur J Appl Physiol. 2015;115:1513–22.

34. Dolezal BA, Potteiger JA. Concurrent resistance and endurance training influence basal metabolic

rate in nondieting individuals. J Appl Physiol (1985). 1998;85:695–700.

35. Dudley GA, Djamil R. Incompatibility of endurance- and strength-training modes of exercise. J Appl Physiol (1985). 1985;59:1446–51.

36. Ferrauti A, Bergermann M, Fernandez-Fernandez J. Effects of a concurrent strength and endurance training on running performance and running economy in recreational marathon runners. J Strength Cond Res. 2010;24:2770–8.

37. Filipas L, Bonato M, Maggio A, Gallo G, Codella R. Effects of plyometric training on different 8-week training intensity distributions in well-trained endurance runners. Scand J Med Sci Sports. 2023;33:200–12.

38. Fyfe JJ, Bartlett JD, Hanson ED, Stepto NK, Bishop DJ. Endurance Training Intensity Does Not Mediate Interference to Maximal Lower-Body Strength Gain during Short-Term Concurrent Training. Front Physiol. 2016;7:487.

39. Glowacki SP, Martin SE, Maurer A, Baek W, Green JS, Crouse SF. Effects of resistance, endurance, and concurrent exercise on training outcomes in men. Med Sci Sports Exerc. 2004;36:2119–27.

40. Gómez-Molina J, Ogueta-Alday A, Camara J, Stickley C, García-López J. Effect of 8 weeks of concurrent plyometric and running training on spatiotemporal and physiological variables of novice runners. Eur J Sport Sci. 2018;18:162–9.

41. Gonçalves R, Motta-Santos D, Szmuchrowski L, Couto B, Soares YM, Damasceno V de O, et al. Combined training is not superior to strength and aerobic training to mitigate cardiovascular risk in adult healthy men. Biol Sport. 2022;39:727–34.

42. Häkkinen K, Alen M, Kraemer WJ, Gorostiaga E, Izquierdo M, Rusko H, et al. Neuromuscular adaptations during concurrent strength and endurance training versus strength training. Eur J Appl Physiol. 2003;89:42–52.

43. Hausswirth C, Argentin S, Bieuzen F, Le Meur Y, Couturier A, Brisswalter J. Endurance and strength training effects on physiological and muscular parameters during prolonged cycling. J Electromyogr Kinesiol. 2010;20:330–9.

44. Hendrickson NR, Sharp MA, Alemany JA, Walker LA, Harman EA, Spiering BA, et al. Combined resistance and endurance training improves physical capacity and performance on tactical occupational tasks. Eur J Appl Physiol. 2010;109:1197–208.

45. Hickson RC. Interference of strength development by simultaneously training for strength and endurance. Eur J Appl Physiol Occup Physiol. 1980;45:255–63.

46. Izquierdo M, Häkkinen K, Ibáñez J, Kraemer WJ, Gorostiaga EM. Effects of combined resistance and cardiovascular training on strength, power, muscle cross-sectional area, and endurance markers in middle-aged men. Eur J Appl Physiol. 2005;94:70–5.

47. Johnston RE, Quinn TJ, Kertzer R, Vroman NB. Strength Training in Female Distance Runners: Impact on Running Economy. The Journal of Strength & Conditioning Research. 1997;11:224.

48. Kelly CM, Burnett AF, Newton MJ. The effect of strength training on three-kilometer performance in recreational women endurance runners. J Strength Cond Res. 2008;22:396–403.

49. Laird RH, Elmer DJ, Barberio MD, Salom LP, Lee KA, Pascoe DD. Evaluation of Performance Improvements After Either Resistance Training or Sprint Interval-Based Concurrent Training. J Strength Cond Res. 2016;30:3057–65.

50. LeMura LM, von Duvillard SP, Andreacci J, Klebez JM, Chelland SA, Russo J. Lipid and

lipoprotein profiles, cardiovascular fitness, body composition, and diet during and after resistance, aerobic and combination training in young women. Eur J Appl Physiol. 2000;82:451–8.

51. Levin GT, Mcguigan MR, Laursen PB. Effect of concurrent resistance and endurance training on physiologic and performance parameters of well-trained endurance cyclists. J Strength Cond Res. 2009;23:2280–6.

52. Losnegard T, Mikkelsen K, Rønnestad BR, Hallén J, Rud B, Raastad T. The effect of heavy strength training on muscle mass and physical performance in elite cross country skiers. Scand J Med Sci Sports. 2011;21:389–401.

53. Mikkola J, Rusko H, Izquierdo M, Gorostiaga EM, Häkkinen K. Neuromuscular and cardiovascular adaptations during concurrent strength and endurance training in untrained men. Int J Sports Med. 2012;33:702–10.

54. Millet GP, Jaouen B, Borrani F, Candau R. Effects of concurrent endurance and strength training on running economy and .VO(2) kinetics. Med Sci Sports Exerc. 2002;34:1351–9.

55. Mirghani SJ, Alinejad HA, Azarbayjani MA, Mazidi A, Mirghani SA. Influence of strength, endurance and concurrent training on the lipid profile and blood testosterone and cortisol response in young male wrestlers. Baltic Journal of Health and Physical Activity. 2014;6.

56. Nelson AG, Arnall DA, Loy SF, Silvester LJ, Conlee RK. Consequences of combining strength and endurance training regimens. Phys Ther. 1990;70:287–94.

57. Panissa VLG, Fukuda DH, de Oliveira FP, Parmezzani SS, Campos EZ, Rossi FE, et al. Maximum Strength Development and Volume-Load during Concurrent High Intensity Intermittent Training Plus Strength or Strength-Only Training. J Sports Sci Med. 2018;17:623–32.

58. Prieto-González P, Sedlacek J. Effects of Running-Specific Strength Training, Endurance Training, and Concurrent Training on Recreational Endurance Athletes' Performance and Selected Anthropometric Parameters. Int J Environ Res Public Health. 2022;19:10773.

59. Robineau J, Babault N, Piscione J, Lacome M, Bigard AX. Specific Training Effects of Concurrent Aerobic and Strength Exercises Depend on Recovery Duration. J Strength Cond Res. 2016;30:672–83.

60. Robineau J, Lacome M, Piscione J, Bigard X, Babault N. Concurrent Training in Rugby Sevens: Effects of High-Intensity Interval Exercises. Int J Sports Physiol Perform. 2017;12:336–44.

61. Rønnestad BR, Hansen EA, Raastad T. In-season strength maintenance training increases well-trained cyclists' performance. Eur J Appl Physiol. 2010b;110:1269–82.

62. Rønnestad BR, Hansen J, Nygaard H. 10 weeks of heavy strength training improves performance-related measurements in elite cyclists. J Sports Sci. 2017;35:1435–41.

63. Sánchez-Moreno M, Rodríguez-Rosell D, Díaz-Cueli D, Pareja-Blanco F, González-Badillo JJ. Effects of Velocity Loss Threshold Within Resistance Training During Concurrent Training on Endurance and Strength Performance. Int J Sports Physiol Perform. 2021;16:849–57.

64. Shamim B, Devlin BL, Timmins RG, Tofari P, Lee Dow C, Coffey VG, et al. Adaptations to Concurrent Training in Combination with High Protein Availability: A Comparative Trial in Healthy, Recreationally Active Men. Sports Med. 2018;48:2869–83.

65. Shaw BS, Shaw I. Compatibility of concurrent aerobic and resistance training on maximal aerobic capacity in sedentary males. Cardiovasc J Afr. 2009;20:104–6.

66. Silva RF, Cadore EL, Kothe G, Guedes M, Alberton CL, Pinto SS, et al. Concurrent training with different aerobic exercises. Int J Sports Med. 2012;33:627–34.

67. Skovgaard C, Christensen PM, Larsen S, Andersen TR, Thomassen M, Bangsbo J. Concurrent speed endurance and resistance training improves performance, running economy, and muscle NHE1 in moderately trained runners. J Appl Physiol (1985). 2014;117:1097–109.

68. Spiliopoulou P, Zaras N, Methenitis S, Papadimas G, Papadopoulos C, Bogdanis GC, et al. Effect of Concurrent Power Training and High-Intensity Interval Cycling on Muscle Morphology and Performance. J Strength Cond Res. 2021;35:2464–71.

69. Štohanzl M, Baláš J, Draper N. Effects of minimal dose of strength training on running performance in female recreational runners. J Sports Med Phys Fitness. 2018;58:1211–7.

70. Sunde A, Støren O, Bjerkaas M, Larsen MH, Hoff J, Helgerud J. Maximal strength training improves cycling economy in competitive cyclists. J Strength Cond Res. 2010;24:2157–65.

71. Terzis G, Spengos K, Methenitis S, Aagaard P, Karandreas N, Bogdanis G. Early phase interference between low-intensity running and power training in moderately trained females. Eur J Appl Physiol. 2016;116:1063–73.

72. Trowell D, Fox A, Saunders N, Vicenzino B, Bonacci J. Effect of concurrent strength and endurance training on run performance and biomechanics: A randomized controlled trial. Scand J Med Sci Sports. 2022;32:543–58.

73. Tsitkanou S, Spengos K, Stasinaki A-N, Zaras N, Bogdanis G, Papadimas G, et al. Effects of high-intensity interval cycling performed after resistance training on muscle strength and hypertrophy. Scand J Med Sci Sports. 2017;27:1317–27.

74. Vorup J, Tybirk J, Gunnarsson TP, Ravnholt T, Dalsgaard S, Bangsbo J. Effect of speed endurance and strength training on performance, running economy and muscular adaptations in endurance-trained runners. Eur J Appl Physiol. 2016;116:1331–41.
